# Non-targeted screening workflows for gas chromatography–high-resolution mass spectrometry analysis and identification of biomagnifying contaminants in biota samples

**DOI:** 10.1007/s00216-020-03018-4

**Published:** 2020-11-06

**Authors:** Andriy Rebryk, Peter Haglund

**Affiliations:** Department of Chemistry, Chemical Biological Centre (KBC), Umeå University, Linnaeus väg 6, 901 87 Umeå, Sweden

**Keywords:** The Baltic Sea, Anthropogenic hazardous substances, Non-target screening, Lipid removal, GC-QTOF/MS, Biomagnification factor

## Abstract

The health of key species in the Baltic region has been impaired by exposure to anthropogenic hazardous substances (AHSs), which accumulate in organisms and are transferred through food chains. There is, thus, a need for comprehensive characterization of the occurrence and accumulation of AHSs in the ecosystem. In this study, we use a non-target screening (NTS) approach for this purpose. A major challenge in NTS of biological samples is the removal of matrix components such as lipids that may interfere with the detection and identification of compounds of interest. Here, we combine gel permeation chromatography with Florisil^®^ column fractionation to achieve sufficient lipid removal for gas chromatography–high-resolution mass spectrometry analysis using electron ionization (EI) and electron capture negative ion chemical ionization (ECNI). In addition, we present new data processing workflows designed to systematically find and identify frequently occurring and biomagnifying AHSs, including known, emerging, and new contaminants. Using these workflows, we discovered a wide range of contaminants in tissue samples from blue mussels, fish, and marine mammals, and calculated their biomagnification factors (BMFs). Compounds with BMFs above 1 for herring and at least one marine mammal included legacy chlorinated pollutants (polychlorinated biphenyls, DDTs, chloro-benzenes/cyclohexanes, chlordanes, toxaphenes, dieldrin), polybrominated diphenyl ethers (PBDEs), and brominated biphenyls. However, there were also several halogenated natural products (halogenated methoxylated brominated diphenyl ethers, 1′-methyl-1,2′-bipyrroles, 1,1′-dimethyl-2,2′-bipyrroles, and the halogenated monoterpene mixed halogenated compound 1) as well as the novel flame retardant Dechlorane 602 and several polycyclic aromatic hydrocarbons, terpenoids, and steroids. The legacy pollutants exhibited the expected biomagnification behavior, demonstrating the utility of the unguided data processing workflow.

**Supplementary Information:**

The online version contains supplementary material available at 10.1007/s00216-020-03018-4.

**Figure:**
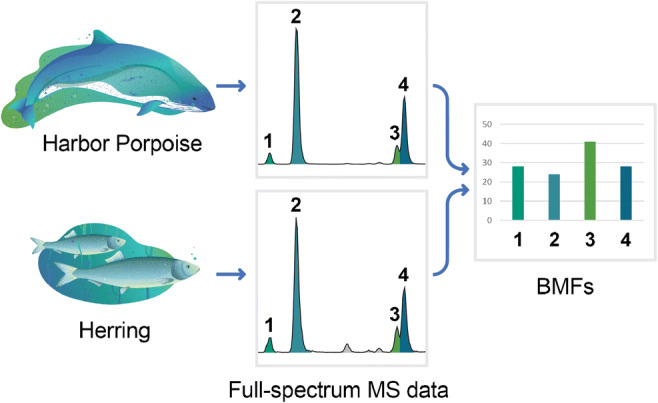
Graphical abstract

## Introduction

Since the onset of industrialization, many chemicals have been designed, synthesized on a large scale, and used in everyday life to meet the needs of mankind. Unfortunately, it was later discovered that many of these chemicals were (and still are) anthropogenic hazardous substances (AHSs) [1]. They are hypothesized, along with other anthropogenic and natural stressors, to be responsible for the extensive population decline of apex predator species in the Baltic Sea [2, 3]. AHSs include but are not limited to compounds such as polychlorinated biphenyls (PCBs), polycyclic aromatic hydrocarbons (PAHs), organochlorine pesticides (OCPs), flame retardants (FRs), pharmaceuticals, and personal care products (PCPs) [1, 2, 4]. AHSs are dangerous to ecosystems because they tend to accumulate in fatty tissues within organisms and to be magnified in organisms at higher trophic levels of the food web as a result of upward transfer from lower levels. Compounds that are persistent, bioaccumulative, and toxic (PBT compounds) have proven to be particularly problematic [2].

**Fig. 1:**
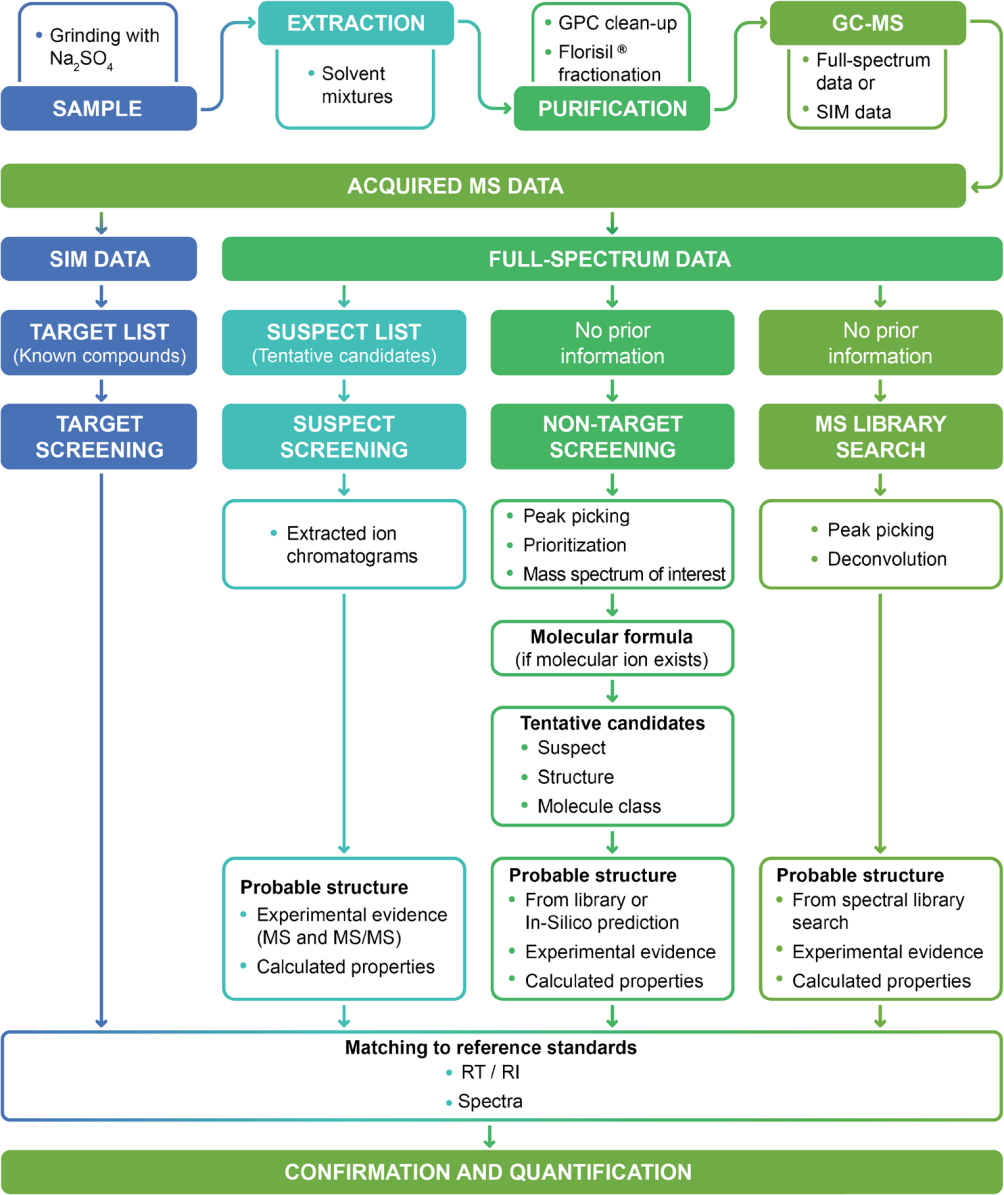
Workflows in target, suspect, and non-target screening (loosely based on Schymanski et al. [11]). GPC, gel permeation chromatography; GC-MS, gas chromatography–mass spectrometry; SIM, selected ion monitoring; MS/MS, tandem mass spectrometry; RT, retention time; RI, retention index

High body burdens of AHSs have been linked to various negative health effects [5] including sterility and reproduction problems in the Baltic seal population and egg shell thinning in birds of prey [6]. International restrictions introduced in the 1970s and 1980s [3, 4] reduced levels of certain AHSs in the environment, and many affected populations recovered. However, the levels of legacy AHSs in the Baltic Sea region have stopped declining rapidly in recent years [3, 6–9]. Furthermore, new industrial chemicals that may potentially be of concern are produced every year [3, 4, 10]. Therefore, it is important to develop tools for assessing exposure to AHSs, the overall health of organisms in the ecosystem, and the state of key physiological processes. This requires a combination of methodologies including spatiotemporal modeling of food web dynamics, analysis of pathogens, and analysis of known, emerging, and new AHSs. The latter is best done using a combination of target screening (TS), suspect screening (SS), and non-target screening (NTS) to determine which hazardous compounds are accumulated in animals’ tissues, how they are transferred through the food web, and what metabolites are formed upon their biodegradation in organisms. As shown in Fig. 1, TS uses reference standards to locate, verify, and quantify analytes; SS is used when reference standards are not available, and relies on the use of prior knowledge to support or reject the presence of a suspect compound in a sample; and NTS is used to detect and quantify other components in samples [11]. NTS methods can be sub-divided into full NTS workflows and library search workflows in which unknown analytes are matched against extensive (spectral) libraries.

In general, untargeted analytical workflows involve non-selective extraction, purification, fractionation (sample preparation), instrumental analysis, and data processing. During sample preparation, it is necessary to extract a wide range of substances of interest while minimizing the amount of co-extracted biogenic materials such as lipids that may influence later stages of analysis. It is difficult to remove lipids completely, but it is often possible to make the lipid:AHS ratio low enough for detection of the latter. Methods for lipid removal include (i) freezing out [12], (ii) sulfuric acid treatment [13], (iii) dialysis [14–16], (iv) adsorption [17–19] and gel permeation chromatography (GPC) [20], (v) solid-phase extraction (SPE) [21], (vi) the “quick, easy, cheap, effective, rugged, and safe” (QuEChERS) method [22], and (vii) other techniques [21].

Existing purification methods give quite good levels of lipid removal, but they all have limitations. For instance, sulfuric acid treatment cannot be applied in NTS because it degrades many AHSs [12], SPE-based lipid removal and QuEChERS can only handle limited sample amounts [23], and GPC can sometimes reduce the lipid content of a sample to a level acceptable for routine analysis [24] but removes only around 90% of a sample’s lipid content. To avoid these problems, one can combine bulk lipid removal (by freezing out, dialysis, or GPC) with adsorption chromatography fractionation. Here, we report the development and application of a two-step process involving GPC clean-up followed by Florisil^®^ column fractionation [17] to obtain AHS-rich purified extracts with low lipid concentrations.

Improvements in hardware, electronics, and data processing have enabled the rapid development of powerful gas chromatography (GC) and liquid chromatography (LC)–high-resolution mass spectrometry (HRMS) instruments such as time-of-flight (TOF) and advanced ion trap (Orbitrap) MS systems. GC-HRMS has been extensively used for target analysis of diverse contaminants in various matrices [25–29], and LC-HRMS is increasingly widely used for suspect and non-target screening [30]. The latter application relies on the ability of TOF and Orbitrap mass analyzers to generate full-spectrum data with high sensitivity, high mass resolution (over 20,000), and good mass accuracy (below 3 ppm) [25, 29, 31]. The availability of accurate high-resolution full-spectrum mass data also enables retrospective analysis and tentative identification of unrestricted numbers of sample constituents because no a priori information about target compounds is needed [11, 25, 31].

Interest in suspect screening and NTS has increased steadily in recent years, and the NORMAN Association has attempted to stimulate and harmonize non-target screening of environmental samples in Europe by hosting a series of workshops [32–34] and a conference [35]. Both the outcomes of these meetings and directed searches of the scientific literature indicate that few suspect screening and NTS studies have been conducted using GC-HRMS. In fact, we found no studies that combined GC-HRMS data acquisition with comprehensive NTS of hazardous substances in marine mammals. However, several NTS studies have used comprehensive two-dimensional GC–electron ionization low-resolution MS (GC×GC-EI-LRMS) [36–42] or electron capture negative ion chemical ionization (ECNI) LRMS [43] to screen for anthropogenic and natural compounds in marine mammals. Furthermore, halogen-specific filters have been applied to HRMS data (excluding the GC dimension) to screen for organohalogens in striped dolphins from the Mediterranean Sea [44].

The aim of this work was to establish an easy-to-use generic workflow for non-selective extraction, purification (lipid removal), and non-target GC-HRMS screening of biological samples, and to use this workflow to identify lipophilic organic contaminants that may threaten top consumers in the Baltic Sea ecosystem. The data evaluation and substance identification efforts were focused on compounds that frequently occur in samples throughout the food web and increase in concentration towards the top, i.e., biomagnifying compounds. Two modes of MS ionization (electron ionization (EI) and ECNI) were used to expand the range of covered contaminants. Biomagnification factors (BMFs) were estimated for many legacy, emerging, and new contaminants in species at low (fish–blue mussels) and high (marine mammals–fish) trophic levels from both benthic and pelagic environments.

## Materials and methods

### Chemicals and reagents

SupraSolv^®^ (for gas chromatography ECD and FID) grade *n*-hexane, dichloromethane (DCM), acetone, diethyl ether, methanol, and isooctane were purchased from Merck KGaA (Darmstadt, Germany); absolute ethanol was purchased from VWR Chemicals (Vienna, Austria); anhydrous sodium sulfate (Na_2_SO_4_) for analysis and Florisil^®^ (0.15–0.25 mm) for column chromatography were purchased from Merck KGaA (Darmstadt, Germany); and a GPC Calibration Solution containing corn oil, bis(2-ethylhexyl)phthalate, pentachlorophenol, perylene, and sulfur was purchased from AccuStandard (New Haven, CT, USA).

### Samples

**Fig. 2:**
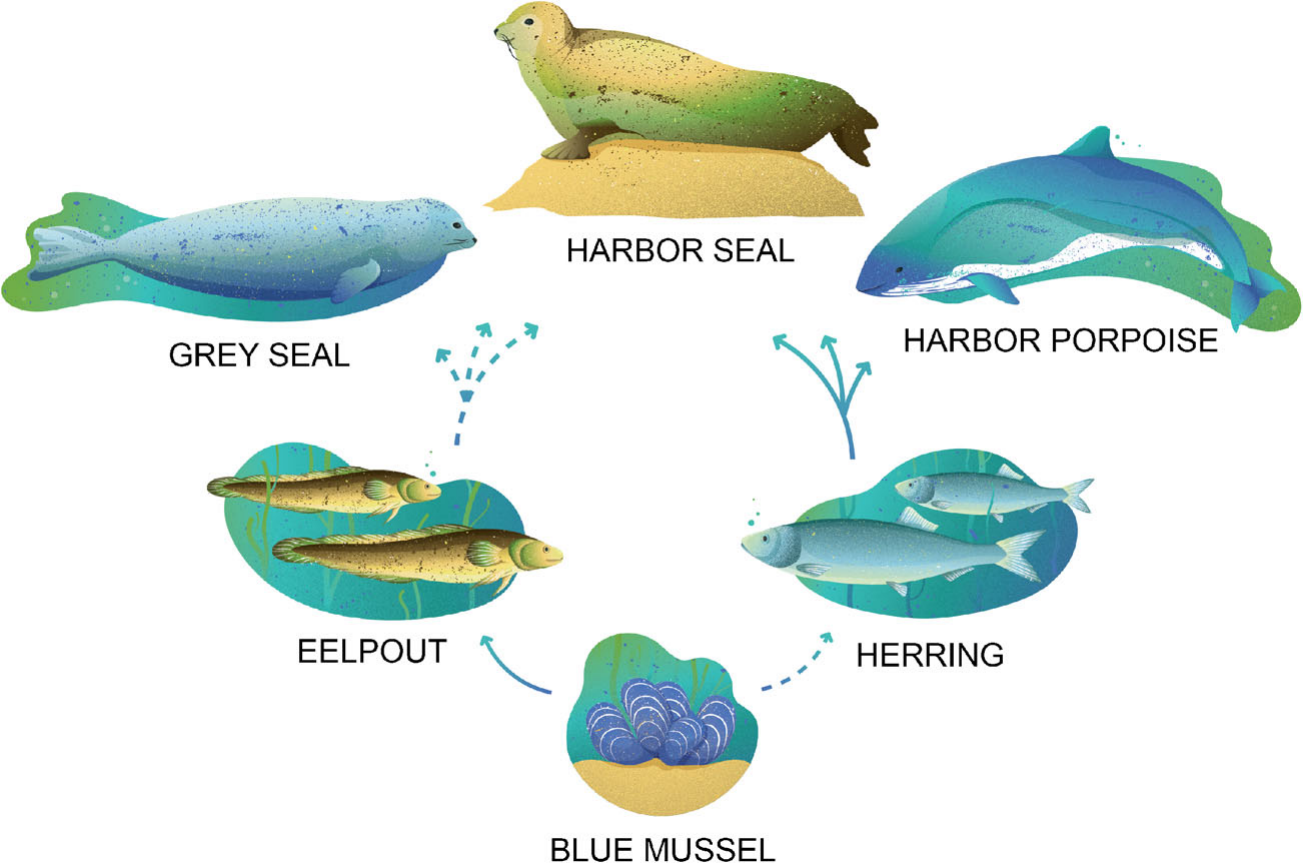
Established predator/prey relationships. Dashed arrows indicate indirect transfer of contaminants

A conceptual food web model of the Baltic Sea ecosystem was created and used to select key benthic and pelagic species for investigation (Fig. 2), focusing on species with high ecological relevance and species with health problems. The following predator/prey relationships were established and investigated: eelpout feeds on blue mussels (lowest level consumer) and three marine mammal species (top consumers) feed on herring. While herring spend part of the year in the coastal zone and may feed on mussel larvae and seals feed on eelpout to some extent, there is no strong coupling between the benthic and pelagic food webs. However, to evaluate contaminant flux, it is important to cover both environments. Eelpout and blue mussels live in the coastal zone, i.e., closer to the source of contaminants, and will exhibit a *fresh* contaminant profile, whereas herring and mammals live in the outer archipelago and open sea and will exhibit an *aged* contaminant profile. This increases the chances of finding and following both semi-persistent/bioaccumulative and highly persistent/bioaccumulative chemicals in the Baltic Sea area.

Samples were collected and prepared by the Swedish Museum of Natural History, Stockholm, which holds permits for Baltic Sea biota sampling and banking granted by the regional ethical review board in Stockholm. In this method development study, we used pooled samples (Table 1) to enhance representativeness. For top consumers, multiple tissues with varying fat contents and metabolic activity were sampled.

**Table 1:** Detailed information on samples used in this study

Species	Tissue	Sample code	Trophic level	Sampling site	Year(s) of sampling	Sample weight, g	Lipid content, %
Blue mussel	Flesh	BMF	Filter feeder	Kvädöfjärden	2016	20	2.1
Eelpout	Muscle	EM	Benthic fish	Kvädöfjärden	2016	50	0.5
Herring	Liver	HL	Pelagic fish	Utklippan	2016	4.4	7.1
Herring	Muscle	HM	Pelagic fish	Utklippan	2016	40	6.3
Grey seal	Muscle	GSM	Top consuming mammal	Baltic Proper	2006–2010	50	0.5
Harbor seal	Blubber	HSB	Top consuming mammal	Baltic Proper	2009–2017	4.5	82
Harbor seal	Liver	HSL	Top consuming mammal	Baltic Proper	2009–2017	9.4	5.0
Harbor seal	Muscle	HSM	Top consuming mammal	Baltic Proper	2009–2017	49	0.9
Harbor porpoise	Blubber	HPB	Top consuming mammal	Southwestern Baltic Proper	2006–2012	4.4	80
Harbor porpoise	Liver	HPL	Top consuming mammal	Southwestern Baltic Proper	2006–2012	9.7	8.8
Harbor porpoise	Muscle	HPM	Top consuming mammal	Southwestern Baltic Proper	2006–2012	49	2.7

### Extraction, clean-up, and fractionation

Before use, all glassware was cleaned and baked overnight in a muffle furnace at 550 °C; disposable materials were used for each experiment.

Samples were homogenized with anhydrous Na_2_SO_4_ (4:1; w/w) 3 times at 10,000 rpm for 3 s in 1-L polypropylene vessels using a Grindomix GM200 laboratory blender (Retsch, Haan, Germany). Samples were then left to stand for about 2 h before being homogenized again. The resulting mixtures were loaded onto glass extraction columns (i.d. 4.0 cm) and extracted in 2 rounds with 2 solvent mixtures according to Jensen et al. [45]: first with 200 mL *n*-hexane:acetone (1:2.5, v/v), then with 150 mL *n*-hexane:diethyl ether (9:1, v/v). After extraction, the samples were reduced in volume to approx. 5 mL by rotary evaporation (Heidolph Instruments, Schwabach, Germany), then 50 mL of ethanol was added to facilitate removal of residual water and the samples were evaporated until only the lipids remained. The lipid content was determined gravimetrically and varied between 0.5 and 82% (Table 1).

A two-step GPC procedure using cross-linked styrene-divinylbenzene columns was developed to reduce the lipid content. Approximately 1 g of the lipids from fatty samples or all the lipids from lean tissues were dissolved in 2 mL DCM:hexane (1:1; v/v) and subjected to semi-preparative GPC using an Agilent Technologies 1260 Infinity II LC System (Santa Clara, CA, USA) consisting of an autosampler, a quaternary pump, a detector, and a fraction collector. The first GPC clean-up was performed with a 50 mm × 21.2 mm Phenogel guard column (10 μm, 100 Å) and a 300 mm × 21.2 mm Phenogel column (5 μm, 100 Å) (both Phenomenex, Torrance, CA, USA). The whole sample (2 mL) was injected, and the analytes were eluted with an isocratic DCM:hexane solvent mixture (1:1; v/v) for 50 min at a flow rate of 5 mL/min (250 mL in total). The column was previously calibrated with the GPC Calibration Solution: the cutoff line was set after the corn oil peak at 13.8 min. The eluent was directed to waste for the first 13.8 min to eliminate most of the lipids, and contaminant fractions were collected from 13.8 to 50 min. All collected fractions were pooled, and their volume was reduced to about 2 mL. Further lipid removal was achieved using two high-resolution GPC columns: a 7.5 mm × 300 mm PLgel (5 μm, 100 Å) and a 7.5 mm × 300 mm PLgel (5 μm, 50 Å) (both Agilent Technologies, Santa Clara, CA, USA). Analytes were eluted with an isocratic DCM:hexane solvent mixture (1:1; v/v) for 40 min at a flow rate of 1 mL/min (40 mL in total). This column system was also previously calibrated with the same GPC Calibration Solution: the cutoff line was set after the corn oil peak at 22.5 min. Eluate collection thus started at 22.5 min. After the clean-up, the sample was reduced in volume to approx. 1 mL. The calibration of the GPC columns was frequently checked using the GPC Calibration Solution.

The samples were then cleaned up by adsorption chromatography fractionation using a Florisil^®^ column (i.d. 1.0 cm, 8.0 g, deactivated with 1.2% H_2_O (w/w)). This step proved essential to reduce lipid residue concentrations to a level acceptable for GC-EI-MS. Four fractions were collected (*n*-hexane, 38 mL; 15% DCM in *n*-hexane, 34 mL; 50% DCM in *n*-hexane, 54 mL; 8% methanol in DCM, 80 mL) following the protocol of Norstrom et al. [46]. The eluate volumes were reduced to about 5 mL, 2 mL isooctane and a volumetric standard (^13^C_12_-CB-97 and ^13^C_12_-CB-188) were added, and the volumes were further reduced to about 1 mL each. The purified samples were then transferred to GC vials. Method blank samples were run in parallel to the biota samples. All but the last Florisil^®^ fractions were submitted to GC-MS analysis; the 4th fraction contained too much matrix (the last remaining lipids) to be analyzed.

### GC/QTOF-MS

Analyses were performed using an Agilent 7890B GC coupled with an Agilent 7250 QTOF-MS (Santa Clara, CA, USA). The system was fitted with a DB-5MS column (30 m × 0.25 mm i.d. × 0.25 µm film thickness; Agilent Technologies, 275 Santa Clara, CA, USA. The injector was operated at a temperature of 300 °C in pulsed-splitless mode with a 50 psi injection pulse lasting for 0.9 min followed by a 100 mL/min purge flow to split vent at 1 min. The GC oven was initially maintained at 80 °C for 2 min then ramped at 5 °C/min to 300 °C, which was held for 2 min. Helium was used as the carrier gas at a constant flow rate of 1.4 mL/min. The MSD transfer line was held at 300 °C.

Most analyses were performed in EI mode at 70 eV electron energy, 10 μA emission current, and an ion source temperature of 250 °C. Data were stored over the mass range of 48 to 450 amu, at an acquisition rate of 1.00 spectrum/s. The prefilter cutoff mass was set to 45 amu.

Additional runs were done in ECNI mode with methane as reagent gas to detect additional halogenated compounds. The GC conditions were similar to those for the EI analysis, but a higher final oven temperature (325 °C) and a higher transfer line temperature (330 °C) were used to extend the boiling point range. The ion source was operated at 195 eV electron energy, 100 μA emission current, and an ion source temperature of 150 °C. Data were stored over the mass range of 33 to 650 amu, at an acquisition rate of 1.00 spectrum/s. The prefilter cutoff mass was set to 30 amu.

### Data processing

The data evaluation focused on finding compounds that occur in samples throughout the food web and increase in concentration towards the top, i.e., biomagnifying compounds. MassHunter Suite (version 10.0; Agilent Technologies, Santa Clara, CA, USA) was primarily used for this purpose. Spectral library searching was done using the National Institute of Standards and Technology (NIST) 2017 EI mass spectral library (National Institute of Standards and Technology, Gaithersburg, MD, USA). MS Excel functions (Microsoft, Redmond, WA, USA) and Python (version 2.7) scripts (Python Software Foundation, Wilmington, DE, USA) were used to filter and align the data. Additionally, GC-Analyzer (MsMetrix, Utrecht, The Netherlands) was used as a complementary software tool for characterization and identification of halogenated compounds detected in ECNI mode.

**Fig. 3:**
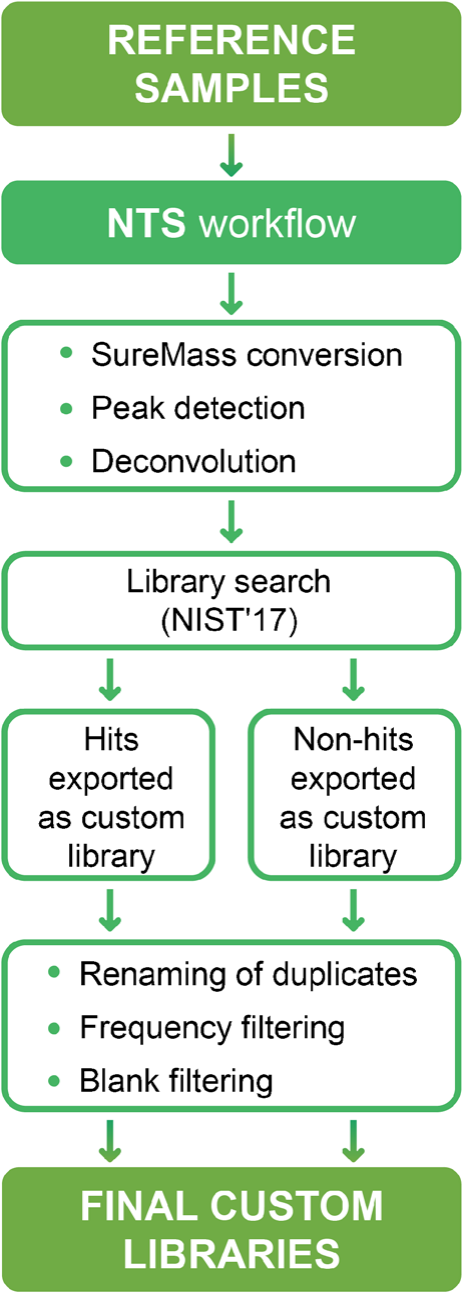
Custom library development workflow. NTS, non-target screening; NIST′17, National Institute of Standards and Technology 2017 EI mass spectral library

**Fig. 4:**
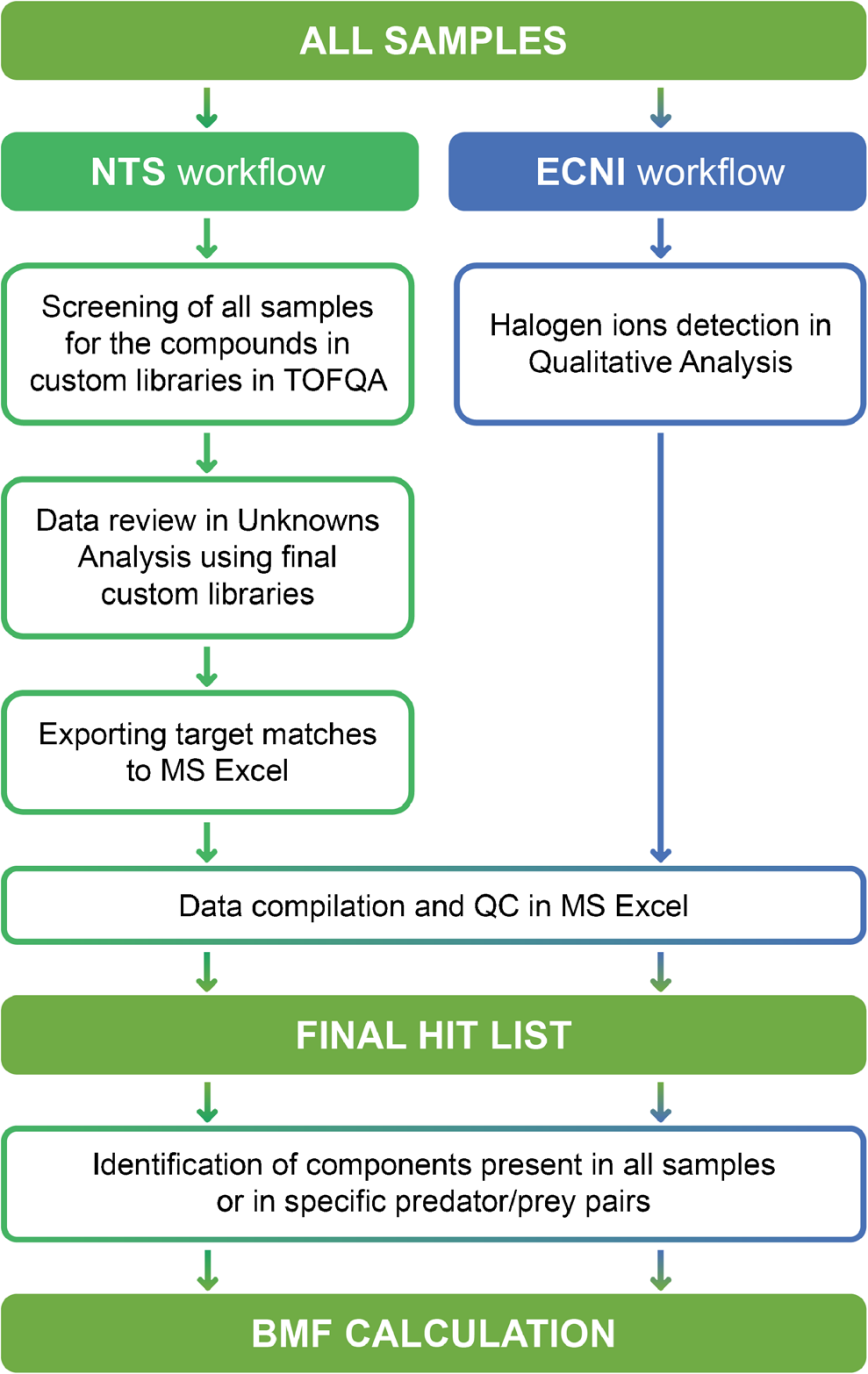
Sample analysis workflows. NTS, non-target screening; ECNI, electron capture negative ionization; QA, MassHunter Quantitative Analysis; QC, quality control; BMF, biomagnification factor

The following data processing steps were performed:

- *Peak picking*. Mass spectrometric analysis in full-spectrum mode generates enormous amounts of information. The first step in data processing is therefore a reduction in data size using an MS-peak picking algorithm. In this step, important information on the mass spectrometric peaks (namely, their area, width, and mass centroid position) is extracted from the raw MS data and stored for further treatment.
- *Non-targeted workflows*. The consensus protocol developed within the NORMAN network was used as a starting point for acquisition and data treatment [11]. A revised version of this workflow is shown in Fig. 1. The non-target screening and library search workflows were combined into one computational pipeline for GC-EI-HRMS data, which was used to create species-specific custom libraries of biota contaminants. The pipeline consists of 2 separate steps, as shown in Figs. 3 and 4.

The first step of this workflow is custom library development, as shown in Fig. 3. Fish samples were chosen as reference (template) samples for this purpose because fish are intermediate links in the food chain and BMFs could therefore only be computed for AHSs present in the fish samples. Data for eelpout muscle and herring muscle samples (reference samples) were processed with Agilent Unknowns Analysis (UA) using a custom UA method, and detected peaks were matched against the NIST 2017 library. Components with sufficiently high spectral similarity (above 60%) were filtered into a “hit list,” assigned tentative names, and exported as custom libraries to Library Editor (LE). The remaining components were filtered into a “non-hit” list, assigned a retention time (RT) identifier, and exported to LE as a second set of custom libraries. Four custom libraries were created in total: herring muscle (HM) “hits” and “non-hits” and eelpout muscle (EM) hits and non-hits. Name assignments were based on spectral similarity; consequently, the same compound name could be assigned to multiple spectra of high similarity. This resulted in duplicates, which would have affected the subsequent data processing and led to inaccurate structure assignments. Therefore, all duplicate features in the EM and HM libraries were renamed using a Python script (see Scripts S1 and S2 in the Electronic Supplementary Material (ESM)) to facilitate further data alignment. The updated custom libraries were then saved.

Frequency filtering was applied to the triplicate reference samples using the custom libraries. Features that were only found in one sample out of three were detected using the VLOOKUP function of MS Excel and then removed from the libraries using another Python script (Scripts S3 and S4 in the ESM), after which the updated versions of the libraries were saved.

Method and solvent blank triplicates were then searched against the new custom libraries; features found in the blanks whose areas exceeded 20% of the sample area were filtered out from all libraries using another Python script (ESM, Scripts S5 and S6), and the remaining components were saved as four final custom libraries: EM hits and non-hits and HM hits and non-hits.

During the second step (Fig. 4), all samples were screened for the components observed in the reference sample custom libraries using TOF Quantitative Analysis software (TOFQA). Separate methods for eelpout muscle and herring muscle were created using the corresponding final custom libraries. Outliers in the reference samples, with deviating qualifier response ratios, due to a bad integration or noisy signal, were scrutinized, and the methods were updated when necessary (see Workflow S1 in the ESM).

This targeted data processing workflow captures components even at signal-to-noise (S/N) ratios as low as 3. However, because data review using TOFQA is time consuming, the results were exported to UA, which offers a better graphical user interface. Additionally, performing the review process in UA makes it possible to look at alternative hits for each “hit” and assign tentative structures to unidentified components (non-hits) present in the spectral libraries that were missed by TOFQA. Components that passed manual review were exported to MS Excel, where the component areas were normalized against the mass of lipids (in grams) originally extracted from the sample components that passed manual review were exported to MS Excel, where the component areas were normalized to 1 g of lipid. The final lists of hits (NIST 2017–based custom libraries) and non-hits were evaluated further, as described below.

#### ECNI workflow

Many known bioaccumulating compounds are halogenated. Therefore, complementary datasets were generated using ECNI and processed specifically for chlorinated and brominated compounds. Extracted ion chromatograms (EICs) were generated for chloride ions (34.9694 Da and 36.9665 Da) and bromide ions (78.9189 Da and 80.9168 Da) in each sample using the Agilent MassHunter Qualitative Analysis (QA) program. The retention times and peak areas of these peaks were exported to MS Excel, where the peaks were aligned, the peak areas were normalized against the mass of lipids (in grams) originally extracted from the relevant sample, and the BMFs were calculated. The sample exhibiting the greatest variation in halogenated compound composition, porpoise blubber, was further processed using QA (manual peak extraction and background subtraction) and MsMetrix GC-Analyzer (automatic peak and spectral deconvolution) to identify as many bioaccumulating halogenated compounds as possible. In several cases, complementary EI spectra were retrieved at the retention times of interesting halogenated compounds to facilitate structure elucidation.

#### Procedure to find and identify new and emerging contaminants

A holistic approach was used to identify potentially biomagnifying AHSs. In this approach, compounds present in all three trophic levels or in specific predator/prey combinations were filtered out, evaluated, and scrutinized to estimate compound-specific BMFs.

Sample constituents of interest were investigated further to gather sufficient information for tentative identification, following the workflows outlined in Fig. 1. Compound names and structures were obtained in several ways: (i) by confirming the names suggested by the NIST 2017 library, (ii) by using retention indices of reference standards (e.g., for PCB identification), and (iii) by manual review, including comparisons of experimental and scientific literature mass spectra and retention indices.

#### Calculation of biomagnification factors

Biomagnification factors were calculated (Eq. (1)) for two predator-prey pairs in the food web: eelpout (benthic fish), which feeds on blue mussels (lowest level consumer), and the three marine mammal species (top consumers), which feed on herring (pelagic fish).

1 $$ BMF=\frac{C(predator)}{C(prey)}\sim \frac{AR(predator)}{AR(prey)} $$

In Eq. (1), *C* denotes the concentration of a given component in a predator or prey sample, and *AR* is the ratio of the component’s area to that of the (closest eluting) volumetric standard, which is proportional to its concentration. Since no quantification standards were used, BMF values were calculated using component area ratios. This approach still yields valid BMFs because the analyte responses are instrument dependent, not sample dependent.

To enhance robustness, BMFs were calculated using the geometric means of the AR values from triplicate GC-MS runs. For compounds that were found in multiple Florisil^®^ fractions, the sum of the ARs was used in the calculations.

## Results and discussion

### Clean-up and fractionation

Both GPC and Florisil^®^ are well tested and widely used clean-up procedures for target analysis of biota samples, which yield high recoveries for a wide range of legacy contaminants [20, 46].

In the present study, the two-step GPC clean-up procedure proved efficient in removing bulk lipids. During the first step, the lipid content of the samples was reduced by 53% on average. The second step reduced the remaining lipid content of the reinjected samples by 86% on average. On average, the two-step GPC clean-up process thus removed 93% of the lipids originally present in the samples. However, the remaining 7% of lipids were still too high to allow reliable NTS.

The cleaned-up samples were therefore subjected to Florisil^®^ fractionation to divide the contaminants and biogenic matrix into sub-groups based on their polarity. Most of the remaining lipids and other polar matrix components were concentrated into the most polar fourth fraction, which was not analyzed in this work. The remaining fractions were found to be relatively clean, and it was possible to analyze batches of samples without peak distortion or background build-up (see ESM Fig. S1).

### Non-target screening

The objective of the NTS workflows was to find sample constituents that were present in all or most of the studied organisms and whose concentration (measured on a lipid weight basis) increased with the trophic level. All components exhibiting these characteristics were reviewed to confirm the structures assigned by the software and to enable additional structure elucidation where needed. This section discusses only contaminants detected in at least 2 trophic levels, but it should be noted that some compounds of potential interest may be present at levels above the limit of detection in samples from certain top consumers (e.g., harbor porpoise) and at levels below the limit of detection in, e.g., fish. Such compounds are, however, at least in part, covered by the halogen selective workflow described in the following section.

PCBs and other legacy pollutants dominated the samples, as expected, and made the discovery of new and emerging contaminants difficult. The final volume of the sample extracts had to be adjusted to keep these abundant contaminants within the dynamic range of the instrument, which resulted in low S/N ratios and moderate spectral quality for other contaminants.

The compounds detected using the non-target screening workflow are listed in Tables 2 and 3, and major contaminant classes are presented in the following section. The structures were confirmed by comparing EI spectra and retention indices and/or by manual review of spectra.

**Table 2:** Biomagnification factors (BMFs) for the eelpout:blue mussel (EM) pair and peak area ratios (normalized against the lipid sample mass in grams) for the herring muscle:blue mussel (HM) and herring liver:blue mussel (HL) pairs for features detected in one or more of the three Florisil^®^ fractions

RT, min	LRI	Comments about compound	Detected in fraction	BMF/area ratio		
				EM	HM	HL
7.21	1147	C_13_H_24_ fragment	Fr. 1	*1.1*	*1.4*	*1.9*
8.00	1175	Bornyl chloride (toxaphene)	Fr. 1	*1.1*	*2.1*	*2.4*
9.09	1214	C_11_H_19_ fragment; possibly terpenoid	Fr. 1	0.8	0.9	*1.2*
9.73	1237	C_11_H_18_O fragment	Fr. 3	0.03	0.03	#N/A*
9.77	1239	C_17_H_30_O/C_12_H_20_O_2_ fragment	Fr. 3	0.1	0.1	0.1
10.11	1251	7a-Methyl-3-(2-methylpropyl)-1,2,4,5,6,7-hexahydroindene	Fr. 1	1.0	0.9	*1.3*
10.18	1254	C_14_H_25_ fragment; possibly terpenoid	Fr. 1	1.0	*1.1*	*1.4*
10.35	1260	C_15_H_28_ fragment; possibly terpenoid	Fr. 1	0.9	0.9	*1.3*
10.54	1267	C_12_H_21_ fragment; possibly terpenoid	Fr. 1	0.9	0.9	*1.3*
10.66	1271	C_15_H_28_ fragment; possibly terpenoid	Fr. 1	*1.5*	*1.3*	*1.8*
10.91	1281	C_9_H_15_ fragment	Fr. 1	*1.1*	0.9	*1.5*
10.93	1282	Unknown composition	Fr. 1	0.8	0.9	1.0
10.99	1284	C_15_H_28_ fragment; possibly terpenoid	Fr. 1	0.9	0.9	*1.7*
11.09	1288	C_15_H_30_ fragment	Fr. 1	1.0	0.8	*1.1*
11.17	1291	C_15_H_28_ fragment; possibly decahydro-1,1,4a,5,6-pentamethylnaphthalene	Fr. 1	1.0	0.9	*1.5*
11.29	1295	Unknown composition	Fr. 3	0.2	0.6	0.7
11.30	1295	Unknown composition	Fr. 1	*1.8*	#N/A	*2.7*
11.38	1298	C_15_H_28_ fragment; possibly terpenoid	Fr. 1	0.8	0.9	1.0
11.54	1304	C_15_H_28_ fragment; possibly terpenoid	Fr. 1	0.8	1.0	*1.4*
11.55	1305	C_11_H_19_ fragment; possibly terpenoid	Fr. 1	0.8	0.9	*1.2*
11.63	1308	Unknown composition	Fr. 1	0.6	*1.1*	*2.2*
12.27	1332	Dodecane, 2,6,11-trimethyl-	Fr. 1	0.9	1.0	*1.4*
12.71	1348	Unknown composition	Fr. 1	1.0	*1.5*	*1.2*
13.12	1364	(+)-epi-Bicyclosesquiphellandrene	Fr. 1	1.0	*1.7*	*2.7*
13.69	1386	Unknown composition	Fr. 2	0.4	#N/A	0.1
14.66	1424	2,2,4,4,7,7-Hexamethyl-1,3,3a,5,6,7a-hexahydroindene	Fr. 1	*1.2*	*2.0*	*2.3*
15.87	1473	C_8_H_12_O/C_6_H_8_N_2_O fragments	Fr. 2	*4.2*	*7.1*	0.9
16.15	1484	C_8_H_12_O/C_6_H_8_N_2_O fragments	Fr. 2	*3.1*	*6.2*	0.8
16.66	1505	C_15_H_24_; possibly sesquiterpene	Fr. 1	0.9	*1.9*	*2.9*
16.83	1512	C_15_H_24_; possibly sesquiterpene	Fr. 1	*1.2*	*1.4*	*2.2*
17.15	1525	C_8_H_12_O fragment	Fr. 3	0.03	0.1	#N/A
17.17	1526	1-Methyl-6-methylidene-4-propan-2-yl-2,3,5,7,8,8a-hexahydro-1*H*-naphthalene	Fr. 1	*1.2*	*1.9*	*2.4*
18.63	1587	C_8_H_9_O fragment	Fr. 3	0.02	0.1	0.1
23.03	1783	Phenanthrene	Fr. 2	1.0	0.5	0.2
24.60	1859	Anthracene, 9-ethenyl-	Fr. 2	*1.1*	*8.3*	0.2
25.94	1924	Unknown composition	Fr. 3	0.02	#N/A	0.1
26.27	1941	CB-52/43	Fr. 1	0.4	*7.8*	*4.9*
27.76	2017	Dehydroabietine	Fr. 1	0.3	#N/A	0.5
28.59	2061	Fluoranthene	Fr. 2	0.5	*1.2*	0.2
29.56	2113	CB-101/113	Fr. 1	*2.3*	*36*	*12*
29.58	2114	Pyrene	Fr. 2	*2.8*	*7.0*	1.0
29.77	2125	2,3,3′,4,4′,5,5′-Heptachloro-1′-methyl-1,2′-bipyrrole (Q1)	Fr. 1	0.8	0.3	0.2
30.65	2174	*p*,*p*′-DDE	Fr. 1	*4.7*	*45*	*15*
31.70	2233	CB-149/139	Fr. 1	#N/A	*26*	*7.5*
31.76	2236	C_24_H_34_O_2_ fragment	Fr. 3	0.04	#N/A	0.2
31.86	2242	CB-118	Fr. 1	*6.0*	*23*	*5.3*
32.18	2261	C_20_H_36_NO_2_P fragment	Fr. 3	0.02	#N/A	#N/A
32.65	2288	CB-153/168/132	Fr. 1	*4.1*	*11*	*4.3*
33.60	2344	CB-138/160/158 + co-eluting *p*,*p*′-DDT	Fr. 1	*11*	*26*	*7.9*
36.38	2516	C_28_H_40_O fragment; probably sterol-type compound	Fr. 3	0.04	0.01	0.4
37.00	2556	C_28_H_40_O fragment; probably sterol-type compound	Fr. 3	0.03	#N/A	0.3
37.42	2583	C_28_H_40_O fragment; probably sterol-type compound	Fr. 3	0.02	0.01	0.1
39.93	2753	Sterol-type compound: probably cholesta-3,5-diene	Fr. 1	#N/A	*5.8*	*12*

Values set in italics are above 1

*Feature was not detected in the corresponding sample

**Table 3:** Biomagnification factors (BMFs) of the features detected using the NTS workflow in one or more of the three Florisil^®^ fractions

RT, min	LRI	Comments about compound	Detected in fraction	BMF						
				GSM	HSM	HSL	HSB	HPM	HPL	HPB
7.21	1147	C_13_H_24_ fragment	Fr. 1	*1.1*	*1.3*	*1.1*	0.8	0.8	*1.3*	0.4
8.00	1175	Bornyl chloride (toxaphene)	Fr. 1	*1.3*	*1.3*	*1.1*	*1.3*	*1.2*	*1.8*	0.7
8.46	1191	Naphthalene	Fr. 1	#N/A*	0.8	1.0	#N/A	*1.3*	0.8	0.3
9.09	1214	C_11_H_19_ fragment; possibly terpenoid	Fr. 1	*1.4*	*1.4*	*1.1*	0.8	1.0	*1.1*	0.7
10.11	1251	1H-Indene, 2,4,5,6,7,7a-hexahydro-7a-methyl-3-(2-methylpropyl)-	Fr. 1	*1.4*	*1.5*	*1.2*	0.7	*1.1*	*1.4*	0.5
10.18	1254	C_14_H_25_ fragment; possibly terpenoid	Fr. 1	*1.5*	*1.4*	*1.2*	0.8	*1.1*	*1.2*	0.6
10.35	1260	C_15_H_28_ fragment; possibly terpenoid	Fr. 1	*1.5*	*1.4*	*1.3*	1.0	1.0	*1.5*	0.5
10.54	1267	C_12_H_21_ fragment; possibly terpenoid	Fr. 1	*1.5*	*1.6*	*1.3*	0.8	*1.2*	*1.5*	0.6
10.66	1271	C_15_H_28_ fragment; possibly terpenoid	Fr. 1	*1.6*	*1.8*	0.9	*1.1*	*1.3*	*1.9*	0.6
10.91	1281	C_9_H_15_ fragment	Fr. 1	*1.6*	*1.3*	*1.4*	*1.1*	*1.2*	*1.7*	0.7
10.93	1282	Unknown composition	Fr. 1	*1.6*	*1.7*	#N/A	#N/A	*1.3*	*1.4*	0.7
10.99	1284	C_15_H_28_ fragment; possibly terpenoid	Fr. 1	*1.5*	*1.4*	*1.2*	0.9	*1.3*	#N/A	0.6
11.00	1288	C_15_H_30_ fragment	Fr. 1	*1.7*	*1.3*	*1.3*	0.9	*1.1*	*1.7*	0.5
11.17	1291	C_15_H_28_ fragment; possibly decahydro-1,1,4a,5,6-pentamethylnaphthalene	Fr. 1	*1.6*	*1.8*	*1.3*	#N/A	*1.2*	*1.6*	#N/A
11.22	1292	C_12_H_18_O fragment	Fr. 1	#N/A	0.6	*1.8*	*2.4*	0.7	1.0	0.2
11.29	1295	Unknown composition	Fr. 1	#N/A	#N/A	0.1	0.8	0.2	0.1	0.1
11.29	1295	Unknown composition	Fr. 3	0.03	0.1	0.02	0.5	0.4	0.1	0.1
11.38	1298	C_15_H_28_ fragment; possibly terpenoid	Fr. 1	*1.5*	*1.6*	1.0	0.8	*1.2*	*1.7*	0.6
11.54	1304	C_15_H_28_ fragment; possibly terpenoid	Fr. 1	*1.4*	*1.4*	*1.1*	*1.2*	*1.1*	*1.5*	0.6
11.55	1305	C_11_H_19_ fragment; possibly terpenoid	Fr. 1	*1.5*	*1.3*	*1.5*	*1.3*	*1.4*	#N/A	#N/A
12.27	1332	Dodecane, 2,6,11-trimethyl-	Fr. 1	*1.4*	*1.4*	*1.2*	0.9	*1.3*	*1.6*	0.6
12.71	1348	Unknown composition	Fr. 1	0.5	0.9	0.5	0.4	0.6	1.0	0.6
13.12	1364	(+)-epi-Bicyclosesquiphellandrene	Fr. 1	*1.4*	*1.4*	*1.2*	*1.2*	*1.1*	*2.0*	0.7
13.85	1392	C_10_H_15_ fragment	Fr. 3	0.3	0.7	0.4	*1.2*	*1.7*	*1.5*	*2.1*
14.02	1399	2-Methylbiphenyl	Fr. 1	0.3	0.4	0.6	*1.8*	0.7	0.5	0.5
14.60	1422	C_14_H_22_ fragment	Fr. 3	#N/A	#N/A	0.6	0.8	*1.7*	*1.9*	*1.9*
14.66	1424	1H-Indene, octahydro-2,2,4,4,7,7-hexamethyl-, *trans*-	Fr. 1	0.8	0.9	0.8	*1.6*	0.7	*1.1*	#N/A
16.25	1488	3-Methylbiphenyl	Fr. 1	0.3	0.5	0.6	*2.3*	1.0	0.8	0.8
16.49	1498	4-Methylbiphenyl	Fr. 1	0.3	0.5	0.7	*1.9*	0.7	0.6	0.6
16.66	1505	C_15_H_24_; possibly sesquiterpene	Fr. 1	*1.2*	*1.3*	0.9	1.0	#N/A	#N/A	0.6
16.83	1512	C_15_H_24_; possibly sesquiterpene	Fr. 1	*1.2*	*1.4*	1.0	0.8	*1.4*	*2.0*	0.7
17.17	1526	1-Methyl-6-methylidene-4-propan-2-yl-2,3,5,7,8,8a-hexahydro-1*H*-naphthalene	Fr. 1	*1.1*	*1.2*	*1.1*	*1.2*	*1.4*	*1.5*	0.5
17.17	1526	C_15_H_24_ fragment; possibly sesquiterpene derivative	Fr. 3	0.3	0.4	0.2	0.4	*1.5*	*1.1*	*1.1*
17.76	1550	Decahydro-8a-ethyl-1,1,4a,6-tetramethylnaphthalene	Fr. 1	*1.4*	*1.5*	*1.3*	*2.2*	*1.4*	*1.6*	0.9
18.63	1587	C_8_H_9_O fragment	Fr. 1	#N/A	#N/A	0.1	0.8	0.3	0.3	0.1
20.58	1672	Bis(2-ethylhexyl) methylphosphonate	Fr. 2	#N/A	#N/A	0.2	*1.3*	0.6	0.6	*6.0*
21.07	1694	Hexachlorobenzene; HCB	Fr. 1	0.1	0.1	0.03	0.1	*2.3*	*4.1*	*1.6*
22.19	1745	Lindane; γ-HCH	Fr. 2	0.7	0.5	0.2	*8.6*	*2.0*	*1.6*	*16.4*
23.03	1784	Phenanthrene	Fr. 1 and 2	0.7	0.5	0.7	*1.4*	0.4	0.5	0.3
24.91	1873	CB-28/31	Fr. 1	0.1	0.1	#N/A	0.3	0.6	0.5	0.6
26.27	1941	CB-52/43	Fr. 1	*1.1*	0.6	0.6	*2.4*	*6.2*	*5.7*	*7.1*
26.41	1948	CB-49	Fr. 1	0.3	0.1	0.2	*1.1*	*3.8*	*4.7*	*3.8*
26.50	1952	CB-48/47/75	Fr. 1	*3.5*	*3.2*	*2.0*	*12*	*13*	*12*	*11*
27.93	2026	Octachlorostyrene	Fr. 1	0.3	0.3	#N/A	*1.2*	0.6	*1.1*	0.9
28.41	2052	CB-74	Fr. 1	0.8	0.9	0.4	*3.2*	*3.3*	*3.5*	*2.9*
28.59	2061	Fluoranthene	Fr. 2	0.2	0.3	0.2	0.8	0.1	#N/A	#N/A
28.64	2064	CB-95/121/88	Fr. 1	0.3	0.2	0.2	0.7	*6.5*	*7.3*	*8.2*
29.41	2105	*p*,*p*′-DDMU	Fr. 1 and 2	0.1	0.1	0.4	*1.4*	0.7	*1.3*	*4.2*
29.56	2113	CB-101/113	Fr. 1	*1.4*	0.7	0.6	*3.6*	*4.1*	*4.7*	*4.6*
29.58	2114	Pyrene	Fr. 2	#N/A	#N/A	0.3	*2.4*	0.3	0.2	#N/A
29.68	2120	*cis*-Chlordane	Fr. 2	#N/A	#N/A	#N/A	*1.2*	#N/A	#N/A	*4.3*
29.71	2122	CB-99	Fr. 1	#N/A	*8.5*	*5.1*	*35*	*12*	*14*	*14*
29.77	2125	2,3,3′,4,4′,5,5′-Heptachloro-1′-methyl-1,2′-bipyrrole (Q1)	Fr. 1	0.003	0.05	0.04	0.6	*14*	*12*	*22*
29.79	2126	*trans*-Nonachlor	Fr. 1 and 2	*1.3*	*1.4*	*1.5*	*14*	*7.7*	*8.8*	*13*
30.14	2145	DDT metabolite with the same formula as DDMS	Fr. 2	#N/A	0.4	*1.1*	*3.1*	*1.1*	*2.0*	*12*
30.27	2152	*p*,*p*′-DDMS	Fr. 2	#N/A	#N/A	0.2	0.9	*1.6*	0.8	*5.0*
30.65	2174	*p*,*p*′-DDE	Fr. 1 and 2	*2.4*	*2.9*	*2.3*	*23*	*3.6*	*4.8*	*9.0*
30.70	2176	Dieldrin	Fr. 3	#N/A	1.0	*1.8*	*3.2*	*2.2*	*9.8*	*9.9*
30.84	2185	CB-110/154	Fr. 1 and 2	0.1	0.2	0.4	0.5	0.6	0.5	0.5
31.10	2199	4,6′-Biazulenyl	Fr. 2	#N/A	#N/A	0.9	*6.3*	*2.3*	#N/A	*15*
31.26	2208	CB-151	Fr. 1	#N/A	0.4	*1.5*	*1.9*	*13*	*16*	*14*
31.40	2215	CB-135/144	Fr. 1	#N/A	0.4	#N/A	*2.4*	*13*	*14*	*16*
31.86	2242	CB-118	Fr. 1	0.4	0.6	0.3	*1.5*	*6.9*	*8.3*	*6.9*
31.90	2244	9-Phenyl-9*H*-fluorene	Fr. 2	#N/A	#N/A	#N/A	0.4	0.1	#N/A	0.7
32.23	2263	*p*,*p*′-DDD + co-eluting *o*,*p*′-DDT (minor constituent)	Fr. 1 and 2	0.4	0.3	0.8	*1.3*	*2.0*	1.0	*5.4*
32.41	2274	CB-146/161	Fr. 1	*39*	*18*	*14*	*67*	*24*	*34*	*28*
32.65	2288	CB-153/168/132	Fr. 1	*41*	*14*	*10*	*59*	*21*	*26*	*24*
32.77	2295	CB-105	Fr. 1	0.8	0.8	0.4	*2.4*	*5.3*	*7.2*	*5.8*
33.54	2340	CB-163/164	Fr. 1	*50*	*21*	#N/A	*80*	*34*	*32*	*41*
33.60	2344	CB-138/160/158 + co-eluting *p*,*p*′-DDT	Fr. 1	*38*	*17*	*11*	*66*	*23*	*28*	*28*
33.82	2357	CB-178	Fr. 1	*34*	*20*	#N/A	*78*	*41*	*46*	*38*
34.13	2376	CB-187/182	Fr. 1	*23*	*16*	*85*	*55*	*30*	*36*	*30*
34.54	2401	CB-128	Fr. 1	#N/A	*11*	*7.5*	*44*	*14*	*16*	*16*
35.43	2456	CB-156	Fr. 1	*7.6*	*3.3*	*2.1*	*17*	*3.5*	*5.4*	*2.7*
36.00	2492	CB-180/193	Fr. 1	*83*	*27*	*14*	*97*	*34*	*39*	*33*
36.38	2516	C_28_H_40_O fragment; probably sterol-type compound	Fr. 3	*1.5*	*4.3*	*2.7*	#N/A	*21*	*4.9*	*5.0*
36.97	2554	CB-170/190	Fr. 1	*81*	*39*	*12*	*104*	*40*	*46*	*40*
37.42	2583	C_28_H_40_O fragment; probably sterol-type compound	Fr. 3	1.0	*2.7*	0.6	#N/A	*6.2*	#N/A	*3.0*
39.93	2753	Sterol-type compound: probably cholesta-3,5-diene	Fr. 1	0.3	0.1	0.3	0.1	0.5	*1.2*	#N/A

BMFs were calculated for the following predator:prey pairs, herring muscle:grey seal muscle (GSM), harbor seal muscle (HSM), harbor seal liver (HSL), harbor seal blubber (HSB), harbor porpoise muscle (HPM), harbor porpoise liver (HPL), and harbor porpoise blubber (HPB). Values set in italics indicate BMFs above 1

*Feature was not detected in the corresponding sample

#### PCBs

Many of the 209 PCB congeners were detected in the herring muscle sample as well as in the top consumer samples (e.g., harbor porpoise blubber), and ~ 50 were detected in all species included in the food chain examined in this study (Tables 2 and 3). Specific congener assignment was done using a database of retention indices generated in-house using authentic reference standards.

#### DDT and its metabolites

The most abundant members of this group of legacy POPs were identified, including *p*,*p*′-DDE, *p*,*p*′-DDT, *o*,*p*′-DDT, and *p*,*p*′-DDMU.

#### Other chlorinated pesticide components

Several other chlorinated contaminants were detected and tentatively identified in the studied samples, including bornyl chloride (1*R*,2*S*,4*R*-2-chloro-1,7,7-trimethylbicyclo[2.2.1]heptane), hexachlorobenzene (HCB), octachlorostyrene, *trans*-nonachlor, lindane, *cis*-chlordane, and dieldrin.

#### PACs

2-Methylbiphenyl, 3-methylbiphenyl, 4-methylbiphenyl, naphthalene, fluorene, phenanthrene, 9-ethenyl-anthracene, fluoranthene, pyrene, 4,5-dihydro-acephenanthrylene, 9-phenyl-9*H*-fluorene, and 4,6′-biazulenyl were tentatively identified.

#### Polycyclic biogenic compounds

Dehydroabietine, 7a-methyl-3-(2-methylpropyl)-1,2,4,5,6,7-hexahydroindene, (+)-epi-bicyclosesquiphellandrene, 2,2,4,4,7,7-hexamethyl-1,3,3a,5,6,7a-hexahydroindene, 1-methyl-6-methylidene-4-propan-2-yl-2,3,5,7,8,8a-hexahydro-1*H*-naphthalene, and hexa-hydro-8a-ethyl-1,1,4a,6-tetramethylnaphthalene were tentatively identified in a similar manner to the PACs.

#### Halogenated natural products

Two halogenated natural products, 2,4,6-tribromoanisole (TBA) and 2,3,3′,4,4′,5,5′-heptachloro-1′-methyl-1,2′-bipyrrole (Q1), were tentatively identified.

#### Partially characterized biogenic compounds

Many of the compounds that were found in both fish and marine mammals could not be well characterized. However, most of these compounds appeared to be hydrocarbons that fragment extensively. Formula generation suggested that the largest fragments of those compounds were generally in the C_10_–C_15_ range, and NIST library searches often returned sesquiterpenes (C_15_H_24_) and related structures. Thus, at least some of these compounds may be terpenoid natural products.

#### Tentative identified laboratory contaminants

We frequently detected and tentatively identified a series of 1*H*-perfluoroalkanes and a number of antioxidant compounds including 4-methyl-2,6-bis(2-methyl-2-propanyl)phenol (butylated hydroxytoluene), 2,4-bis(2-methyl-2-propanyl)phenol, and 2,4-*ditert*-butyl-6-(2,4-*ditert*-butyl-5-hydroxyphenyl)phenol in both samples and blanks. The fluorinated contaminant was traced to the GPC clean-up step and probably originates from the Teflon tubing used in the apparatus. It was not detected in the extraction blank but appeared in all blanks created after the GPC steps. The antioxidants are probably solvent impurities.

### Halogen selective screening

The ECNI workflow revealed many *brominated compounds* that were overlooked during NTS processing of the EI data. This may be partially due to the high sensitivity and specificity of ECNI bromide ion detection [47]. Thus, polybrominated diphenyl ethers (PBDEs) and other compounds of potential interest, which are present in the samples at much lower levels than PCBs, may have been missed during peak picking based on EI data but were easily found in the EICs of the bromide ion. The improved detection of brominated compounds by ECNI could also be partly due to the effective elimination of detector responses originating from non-halogenated compounds, which may give rise to chimeric EI spectra in the event of partial or severe co-elution.

**Fig. 5:**
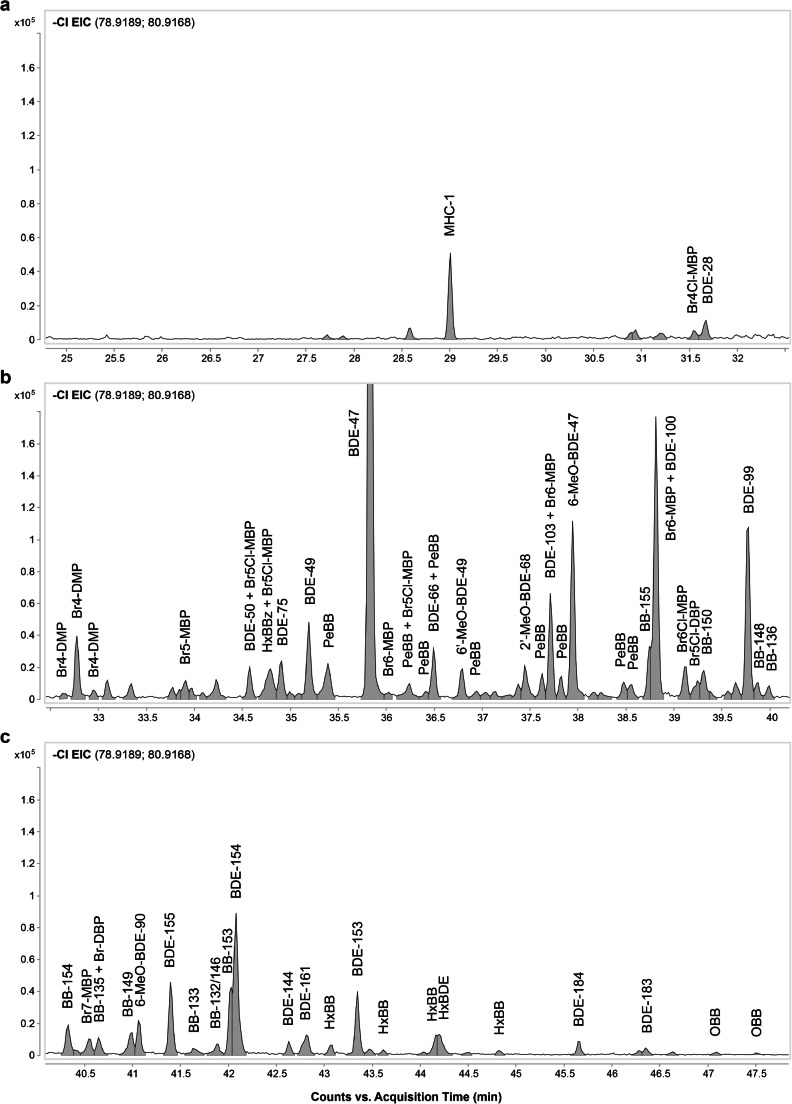
(**a**–**c**) Extracted ion chromatograms of bromide ions from ECNI analysis of porpoise blubber, Florisil^®^ fraction 1, with names of tentatively identified compounds indicated. BB, brominated biphenyl; BBz, brominated benzene; BDE, brominated diphenyl ether; MeO, methoxy; MHC, mixed halogenated compound; MBP, methyl-bipyrrole; DBP, dimethyl-bipyrrole

The identification of the brominated compounds was primarily performed using the porpoise blubber sample. Full ECNI spectrum was extracted at the retention times of the bromine EIC (m/z 79/81) peaks and carefully examined to find molecular or high molecular weight fragment ions. If no such ions were found, a background-subtracted EI spectrum was extracted at the same retention time. Using molecular ion and fragment ion formula generation, manual spectra interpretation, and spectra and retention indices generated in-house or extracted from the scientific literature (see references in the following sections), tentative structures could be assigned to most of the bromine EIC peaks in the porpoise blubber (see Fig. 5). The major contaminant classes identified in this way are described below.

#### PBDEs and polybrominated biphenyls

The tentatively identified brominated compounds include several PBDE congeners (BDEs 28, 47, 49, 50, 66, 99, 100, 103, 153, 154, 155, 161, 183, and 184) and several polybrominated biphenyl (PBB) congeners (nine PeBBs; HxBBs 132, 133, 135, 136, 146, 148, 149, 150, 154, and 155; and two OBBs). Peak assignments for these compounds were based on manual spectral interpretation, an in-house spectral and retention index database, and literature data [48–50].

#### Halogenated natural products

In addition to the anthropogenic brominated compounds, several brominated, chlorinated, and mixed halogenated natural products (HNPs) were detected. These included methoxylated BDEs (MeO-BDEs) [51, 52], mixed halogenated compound 1 (MHC-1; monoterpene; C_10_H_13_Br_2_Cl_3_) [53], Q1 (2,3,3′,4,4′,5,5′-heptachloro-1′-methyl-1,2′-bipyrrole) [54], several brominated and mixed halogenated 1′-methyl-1,2′-bipyrroles [55], and several brominated and mixed halogenated 1,1′-dimethyl-2,2′-bipyrroles [56].

A few HNPs were detected in samples from different trophic levels, allowing BMFs to be calculated. These included Q1, MHC-1, four MeO-PBDEs (6-MeO-BDE-47, 6-MeO-BDE-49, 2′-MeO-BDE-68, and 6-MeO-BDE-90), two MBPs (Br_5_Cl-MBP and Br_6_-MBP), and two DBPs (Br_4_Cl-DBP and Br_5_Cl-DBP). For full information on the HNPs detected and identified in species at different trophic levels, see Tables 2, 3, and 4.

**Table 4:** Biomagnification factors (BMFs) of the brominated features detected using the ECNI workflow in one or more of the three Florisil^®^ fractions

RT, min	LRI	Comments about compound	Detected in fraction	BMF						
				GSM	HSM	HSL	HSB	HPM	HPL	HPB
18.85	1596	2,4,6-Tribromoanisole (TBA)	Fr. 2	0.005	#N/A*	0.01	#N/A	0.1	#N/A	#N/A
28.41	2052	Unknown composition	Fr. 1	0.7	#N/A	#N/A	0.3	*2.0*	1.0	#N/A
29.02	2084	MHC-1	Fr. 2	#N/A	#N/A	#N/A	0.7	*3.7*	0.6	*51*
29.46	2108	Q1^#^	Fr. 1	0.01	0.02	0.02	0.1	*10*	*8.0*	*36*
30.81	2182	Parlar 26^#^	Fr. 1	0.01	0.03	0.04	0.2	0.9	0.4	*8.8*
31.51	2222	Br_4_Cl-MBP	Fr. 3	0.4	0.2	0.1	#N/A	0.2	#N/A	#N/A
32.79	2296	Br_4_Cl-DBP	Fr. 1	#N/A	#N/A	#N/A	*3.1*	*8.8*	*9.8*	*23*
33.33	2328	Unknown composition	Fr. 3	#N/A	#N/A	#N/A	0.2	0.9	*2.5*	*3.0*
34.02	2369	Parlar 50^#^	Fr. 1	0.01	0.01	0.02	0.2	0.9	0.1	*17*
34.59	2404	BDE-50 + Br_5_Cl-MBP	Fr. 1	#N/A	#N/A	#N/A	0.2	1.0	*1.1*	*2.5*
34.80	2417	HxBBz + Br_5_Cl-MBP	Fr. 1	1.0	#N/A	#N/A	0.7	*3.2*	*1.5*	*1.3*
35.21	2442	BDE-49	Fr. 1 and 2	#N/A	#N/A	#N/A	0.1	0.6	0.6	*6.6*
35.83	2481	BDE-47 + PeBB	Fr. 1, 2, and 3	0.4	0.5	1.0	*3.4*	*3.5*	*2.2*	*35*
36.30	2511	Br_5_Cl-MBP + PeBB	Fr. 1	#N/A	#N/A	#N/A	#N/A	*2.0*	*1.4*	*3.4*
36.51	2524	BDE-66 + PeBB	Fr. 1, 2, and 3	0.2	#N/A	#N/A	0.9	*3.8*	*3.1*	*49*
36.80	2543	6-MeO-BDE-49	Fr. 2 and 3	0.1	#N/A	#N/A	0.3	*1.1*	0.7	*8.8*
37.47	2587	2′-MeO-BDE-68 + Br_5_Cl-MBP	Fr. 3	0.05	#N/A	0.02	0.9	*2.0*	*2.6*	*4.2*
37.73	2604	BDE-103 + Br_6_-MBP	Fr. 1 and 2	#N/A	#N/A	#N/A	0.9	*2.2*	*2.0*	*25*
37.96	2619	6-MeO-BDE-47	Fr. 2 and 3	0.3	0.2	#N/A	1.0	*5.9*	*4.5*	*87*
38.83	2678	BDE-100 + PBB-155 + Br6-MBP	Fr. 1 and 2	#N/A	0.4	0.1	*3.1*	*5.6*	*4.8*	*54*
39.07	2694	Br_6_Cl-MBP	Fr. 3	#N/A	#N/A	#N/A	0.8	1.0	*1.3*	*3.0*
39.28	2708	Br_5_Cl-DBP	Fr. 1	#N/A	#N/A	#N/A	0.4	*3.6*	*5.1*	*6.6*
39.58	2729	PBB-150 and unknown co-elution	Fr. 2	#N/A	#N/A	#N/A	1.0	*1.5*	0.8	*8.0*
39.63	2732	Dechlorane 602^#^	Fr. 1	*3.7*	*3.0*	*4.1*	*9.7*	*20*	*18*	*16*
39.79	2744	BDE-99	Fr. 1	0.5	#N/A	#N/A	*1.3*	*8.4*	*7.8*	*18*
41.08	2835	6-MeO-BDE-90	Fr. 2	0.3	0.3	0.3	*1.1*	*2.1*	0.9	*9.7*
41.43	2860	BDE-155	Fr. 1	0.5	#N/A	#N/A	*5.1*	*12*	*15*	*13*
42.06	2906	BDE-154 + PBB-153	Fr. 1 and 2	#N/A	0.6	0.5	*3.7*	*3.9*	*3.0*	*40*
42.85	2964	BDE-161	Fr. 1	#N/A	#N/A	#N/A	#N/A	*6.4*	*7.7*	*11.3*
43.35	3002	BDE-153	Fr. 1	*2.5*	*1.3*	0.7	*11*	*23*	*22*	*37*
44.19	3066	Unknown composition	Fr. 3	#N/A	#N/A	#N/A	0.9	0.2	#N/A	*3.9*

BMFs were calculated for the following predator:prey pairs, herring muscle:grey seal muscle (GSM), harbor seal muscle (HSM), harbor seal liver (HSL), harbor seal blubber (HSB), harbor porpoise muscle (HPM), harbor porpoise liver (HPL), and harbor porpoise blubber (HPB). Values set in italics indicate BMFs above 1

*Feature was not detected in the corresponding sample

^#^Chlorinated compound (included as it is discussed in the “Results and discussion” section)

#### DDT metabolites

In addition to the DDT metabolites detected and tentatively identified by EI (*p*,*p*′-DDD, *p*,*p*′-DDE, *p*,*p*′-DDMS, and *p*,*p*′-DDMU), three further metabolites were tentatively identified using the ECNI workflow: *o*,*p*′-DDMU, *p*,*p*′-DDNS, and *p*,*p*′-DDNU.

### Manual investigation of compounds missed by NTS and ECNI workflows

Legacy contaminants such as PCBs, DDTs, and other organochlorine pesticides dominated the EI total ion chromatograms (TICs) and the ECNI chlorine EICs. This makes it difficult to find new and emerging contaminants, as noted before; automated peak picking algorithms can easily miss minor sample constituents that co-elute with legacy contaminants.

**Fig. 6:**
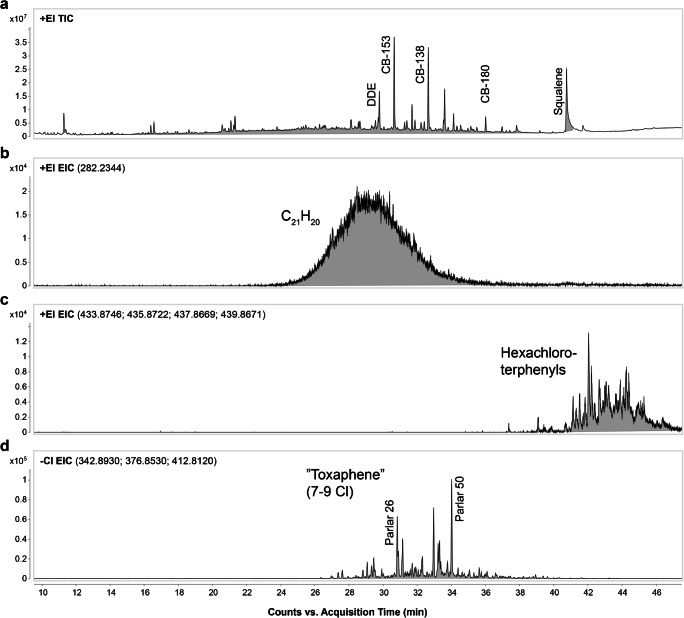
Chromatograms of poorly resolved non-polar compounds (Florisil^®^ fraction 1) in porpoise samples. (**a**) Total ion chromatogram (TIC) from EI analysis of porpoise muscle; unresolved compounds under the baseline between retention times 12–40 min. (**b**) Extracted ion chromatogram (EIC) of ions with formula C_21_H_20_. (**c**) EIC of hexachloroterphenyls. (**d**) EIC for toxaphene-related compounds (hepta-nonachlorobornanes) from ECNI analysis of porpoise blubber

In addition, close inspection of the TICs revealed an elevated base line in the middle of the chromatograms generated by EI analysis of the first Florisil^®^ fractions (see Fig. 6a). Manual extraction of spectra from various regions of the baseline yielded similar spectra with extensive fragmentation and a repetitive pattern spaced by 14 Da (see ESM Fig. S2). Formula generation indicated that these fragments consisted only of carbon and hydrogen and contained many rings or double bonds (5–8 double bond equivalents (DBEs)). EICs of the major fragments exhibited very broad peak envelopes that probably reflected the presence of a plethora of isomeric compounds, as exemplified by the EIC for C_21_H_20_ shown in Fig. 6b. The spectral and retention indices suggest that most of the unresolved hydrocarbons are in the C_18_–C_28_ range. Squalene (C_30_H_50_; DBE 5.5) was identified at the end of the unresolved peak, which may indicate that the unresolved components are non-polar (unsaponifiable) lipids. This high-hydrocarbon background complicated peak detection and deconvolution of peaks and spectra.

A similar inspection of the later part of the TIC (see Fig. 6a) revealed another group of unresolved sample constituents (polychlorinated terphenyls (PCTs)). The combined EIC for the hexa-CTs is shown in Fig. 6c. PCTs are closely related to PCBs but have four more positions open for substitution, resulting in thousands of potential congeners. Such poorly resolved envelopes of isomeric peaks present considerable challenges for peak picking and deconvolution algorithms.

The TIC of the ECNI contained no hydrocarbon signals because such compounds respond poorly in ECNI, but nevertheless featured an elevated baseline. One compound class that contributed to this elevation was toxaphene-related compounds (chlorinated bornanes, bornenes, bornadienes, camphenes, and dihydrocamphenes). A combined EIC of heptachlorobornanes to nonachlorobornanes is shown in Fig. 6d. This envelope is slightly different from that for compounds such as PCTs. At the base, there is a broad peak of unresolved congeners (this compound class has almost 100,000 possible congeners), which is overlaid by a few highly persistent congeners. Peak picking from top consumer ECNI data allowed some of these compounds to be tentatively identified as B7-1001, B7-1450, B8-1413 (Parlar 26), B8-1412, B8-531/1414/1945, B8-806, B8-2229 (Parlar 44), and B9-1679 (Parlar 50) by comparison to literature data [57, 58]. However, the concentrations of these compounds in fish were lower, so the corresponding features were often lost during the data filtering process. This calls for an alternative top-down workflow that would start with comprehensive non-target analysis of top consumer samples and targeted screening of lower trophic level samples.

### Biomagnification factors of tentatively identified compounds

Biomagnification is an increase in the concentration of a contaminant from lower to higher trophic levels in a food chain, and the BMF is the ratio of the concentration of a given contaminant in the predator to that in its prey (after normalization against lipid weight). BMF values > 1 indicate that the contaminant biomagnifies. The BMF concept does not account for metabolic alterations of the contaminants, and a predator generally feeds on more than one prey species. Therefore, BMFs should not be treated as exact values [59].

The estimated BMFs for all workflows and species combinations are listed in Tables 2, 3, and 4. BMF values above 1 are highlighted with a grey background to make it easier to spot potentially bioaccumulating compounds.

Although several compounds were found in both blue mussels and fish, only a few showed any major bioaccumulation potential (Table 2). In the case of the eelpout:blue mussel pair, BMFs above 2 were only estimated for *p*,*p*′-DDE, a few PCB congeners (CB-101/113, CB-149/139, CB-118, CB-153/168/132, and CB-138/160/158), and two unknown compounds, which yield C_6_H_12_O and C_6_H_8_N_2_O fragments but no molecular ions. In the herring:blue mussel pair, a somewhat greater number of compounds had lipid weight–adjusted BMFs above 2; together with the compounds mentioned above, there was one additional PCB (CB-52/43), bornyl chloride, pyrene, 9-ethyl-anthracene, four sesquiterpenes (including (+)-epi-bicyclosesquiphellandrene and 1-methyl-6-methylidene-4-propan-2-yl-2,3,5,7,8,8a-hexahydro-1*H*-naphthalene), and three unknown compounds. The PCB congeners, PAHs, and two unknown compounds (C_6_H_12_O/C_6_H_8_N_2_O fragments; Table 2) seemed to preferentially distribute to herring muscle rather than herring liver. The lipid-adjusted area ratios for the PAHs and the two unknown compounds were clearly above 1 for the herring muscle:blue mussel pair and clearly below 1 for the herring liver:blue mussel pair, indicating that these compounds have limited metabolic stability.

The number of compounds found in the higher trophic level samples using the NTS workflow was around twice that in the lower trophic level samples (Table 3). Most of the tentatively identified compounds with BMFs above 2 were PCBs, OCPs (DDTs, lindane, *cis*-chlordane, *trans*-nonachlor, and dieldrin), or DDT metabolites (*p*,*p*′-DDE, *p*,*p*′-DDMU, and *p*,*p*′-DDMS). The remaining tentatively identified compounds with at least one estimated BMF value above 2 were HCB, three PAHs (3-methylbiphenyl, pyrene, and 4,6′-biazulenyl), one sesquiterpene (decahydro-8a-ethyl-1,1,4a,6-tetramethyl-naphthalene), and three unknowns that may have been steroid derivatives.

The remaining compounds were dominated by terpenoids and low molecular weight PAHs, with estimated BMFs in the range 0.5 to 2. Many of those had BMFs slightly above 1, when comparing top consumer muscle and liver tissue to herring muscle, and slightly below 1, when comparing top consumer blubber to herring muscle, indicating differences in tissue distribution or biotransformation/biosynthesis.

The highest BMFs ranged from 5 to 100 and were generally found for PCBs, OCPs, and DDT metabolites. The BMFs of these legacy POPs were similar across tissue types for the harbor porpoise, but those in harbor seal blubber differed markedly from those in the liver and muscles. This may be explained by the higher metabolic capacity of pinnipeds such as the harbor seal when compared to cetaceans such as the harbor porpoise [60, 61]. The metabolic activity of muscle and liver tissue of harbor seals is higher than that of blubber tissue, leading to lower BMFs in the former.

In accordance with this hypothesis, the highest BMFs for non-legacy POPs were found when comparing top consumer blubber to herring muscle. The following tentatively identified compounds had BMFs above 2 when comparing at least one blubber sample to herring muscle: 3-methylbiphenyl, pyrene, 4,6′-biazulenyl, and decahydro-8a-ethyl-1,1,4a,6-tetramethylnaphthalene. Thus, the three PAHs and the sesquiterpene likely have high biomagnification potential but limited metabolic stability.

The alternative ECNI workflow expanded the range of compounds that could be detected in both herring muscle and top consumer tissues (Table 4). In particular, it was effective in detecting and tentatively identifying brominated compounds. About half of these brominated compounds were PBDEs and PBBs. The remaining compounds were primarily halogenated natural products, specifically TBA, MeO-PBDEs, MHC-1, MBPs, and DBPs.

To our knowledge, this work presents the first reported BMFs for MeO-BDEs, Q1, MHC-1, MBPs, DBPs, and Dechlorane 602 in top consumers in the Baltic Sea food web.

Many BMFs could be calculated for harbor seal blubber vs. herring muscle and for harbor porpoise (all tissue types) vs. herring muscle. On the other hand, many of the brominated compounds were below the limit of detection in grey seal muscle, harbor seal muscle, and harbor seal liver, possibly due to limited resistance to metabolism. The levels of the compounds listed in Table 4 were also below the limit of detection in blue mussels; consequently, no data are shown for the benthic food web.

Most PBDEs and PBBs seem to magnify to a similar extent to PCBs in porpoise blubber, but to a somewhat lesser extent in harbor seal blubber and harbor porpoise muscle and liver. The magnification of the most strongly biomagnifying HNP, 6-MeO-BDE-47, in harbor porpoise tissues is also similar to that of PCBs. MHC-1, Q1, and the two halogenated DBPs (Br_4_Cl-DBP and Br_5_Cl-DBP) exhibit moderate magnification potential in harbor porpoise tissues, with BMFs in the range of 0.6–51, 8.0–36, 8.8–23, and 3.6–6.6, respectively. The halogenated MBPs may also have biomagnification potential, but co-elution made it difficult to determine BMFs from bromine ion EICs.

BMFs were also calculated for two toxaphene-related compounds, Parlar 26 and Parlar 50, and one novel flame retardant, Dechlorane 602, which were manually identified after being missed during automatic data processing. BMFs above 1 were only found for Parlar 26 (BMF = 8.8) and Parlar 50 (BMF = 17) when comparing harbor porpoise blubber to herring muscle. Dechlorane 602 exhibited biomagnification in all top consumer species and tissues; as with the other magnified compounds, the highest BMFs were observed for harbor porpoise tissues (cf. Table 4).

### Comparison of biomagnification factors

This study fills a knowledge gap resulting from a lack of comprehensive systematic studies on bioaccumulating compounds in the Baltic Sea region. In the following section, we discuss the biomagnification of legacy pollutants throughout the Baltic Sea ecosystem and compare the results obtained in this work to BMF values reported for other parts of the world. As far as possible, comparisons are made to studies focusing on the Baltic Sea and other northern waters. For HNPs and emerging contaminants, however, reports from these regions are sparse, so comparisons are made to southern waters.

#### Biomagnification of PCBs and DDTs in the Baltic Sea ecosystem

**Fig. 7:**
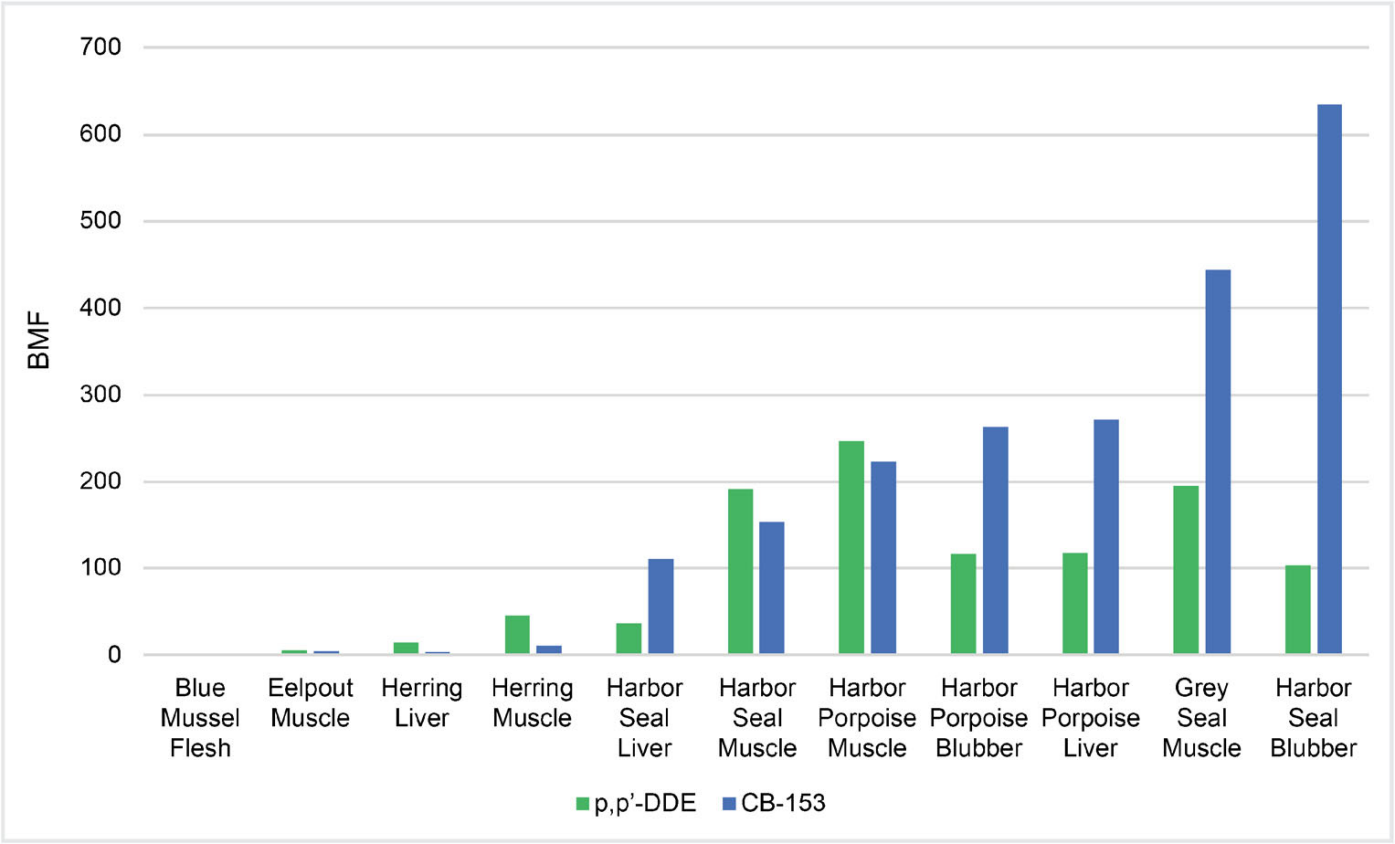
BMFs of two legacy persistent organic pollutants, CB-153 and *p*,*p*′-DDE, in the samples under study

The BMFs of two well-known legacy POPs in various species and tissues are presented in Fig. 7 to illustrate the strong biomagnification occurring in the Baltic Sea ecosystem and the utility of the NTS workflow (BMFs were generated prior to identification of contaminants). The BMF values are all computed relative to the concentrations observed in blue mussels (l.w. basis), which were set to 1 because blue mussels are filter feeders that act as the lowest level consumers in the investigated food web.

#### Legacy contaminants in the Baltic Sea and North Sea regions

Routti et al. [62] reported BMFs for PCBs and DDTs ranging from 30 to 140 in Baltic grey seals. These PCB values agree well with the values reported here (Table 3), which range from 23 to 83 for hexa- and hepta-CBs. However, lower BMFs were obtained for DDT and its metabolites (those for *p*,*p*′-DDE range from 2.2 to 23), possibly because the samples examined here were sampled later than those of Routti et al. [62] and reflected an aged contaminant profile.

Ruus et al. [63] reported BMFs for total PCBs, DDTs, chlordanes (CHLs), hexachlorocyclohexane (HCH), and HCB in blubber of Norwegian grey and harbor seals. The reported values depend on prey and are in the range of 8.7–28 for PCBs, 9.9–37 for DDTs, 10–34 for CHLs, 2.1–2.5 for HCH, and 0.3 for HCB in harbor seals. Similar values were obtained in the current study for HCH (8.6), HCB (0.1), *p*,*p*′-DDE (23), and PCBs (17–80 for hexa-CBs). The BMF for *cis*-chlordane was 1.2, which is slightly lower than the BMF for CHLs reported by Ruus et al. [63].

Weijs et al. [64] reported BMF values in 2009 for PCBs and PBDEs in blubber tissue of adult harbor seals and harbor porpoises from the Southern North Sea. The BMFs for CB-153, BDE-47, and BDE-153 in harbor seals were 65, 4.6, and 15, respectively, which are very similar to those obtained in this work (59, 3.4, and 11, respectively; see Table 4). The BMFs determined by Weijs et al. [64] for these compounds in harbor porpoises were 54, 15, and 77, respectively, which are again similar to those obtained in this work (24, 35, and 37, respectively). However, more recent studies by Shaw et al. [65] indicated higher BMFs for Atlantic harbor seals and Atlantic herring: the BMF of CB-153 was threefold higher than that determined in this work (178 vs. 59), and the BMFs of BDEs were fivefold to tenfold higher (14 to 54 vs. 1.3 to 11) [66].

#### Halogenated natural products in other cold waters

Weijs et al. [67] reported BMF values for naturally produced MeO-PBDEs in blubber tissue of harbor seals and harbor porpoises from the Southern North Sea. The BMF values for 2′-MeO-BDE-68 and 6-MeO-BDE-47 in adult male harbor porpoises were in the range of 0.4–2.0 and 3.6–6.9, respectively [67]. The BMF values for 6-MeO-BDE-47 in adult harbor seals were considerably lower, ranging from 0.1 to 0.3, possibly due to this species’ higher metabolic rates [67]. Similarly, 2′-MeO-BDE-68 and 6-MeO-BDE-47 were reported to biomagnify slightly in a Canadian Arctic marine food web, with trophic magnification factors (TMFs) of 2.3 and 2.6, respectively [68]. The cited BMF values are consistent with those obtained in the current study (Table 4): 4.2 for 2′-MeO-BDE-68 and 87 for 6-MeO-BDE-47 in harbor porpoises, and 1.0 for 6-MeO-BDE-47 in harbor seals.

Tittlemier et al. [69] also studied Canadian Arctic food webs and observed significant biomagnification of four halogenated DBPs in the invertebrate–fish–seabird food web (TMF range 5.2–15), but not in the invertebrate–fish–ringed seal food web, possibly because pinnipeds can metabolize halogenated DBPs. Pangallo and Reddy [70] reported similar biomagnification behavior for halogenated MBPs in a Northwest Atlantic food web. The halogenated MBP concentrations generally increased with the trophic level, but these compounds were not found in pinniped blubber, probably because they were rapidly metabolized. Those observations are consistent with our findings (Table 4). BMFs above 1 were only observed for halogenated DBPs and MBPs in grey seal blubber and harbor porpoise tissue samples, which all have limited metabolic capability.

#### Halogenated natural products and emerging contaminants in warm waters

Several comprehensive studies on AHSs and HNPs have been conducted recently. Many have focused on dolphin species in warm water environments because dolphins are considered suitable top consumer species for biomonitoring and HNPs are abundant in such waters [36].

Several such studies originate from North America. In 2012, Hoh et al. [37] reported the identification of 271 compounds belonging to 24 chemical classes in Atlantic common dolphin (*Delphinus delphis*) blubber. All compounds bar one were halogenated, 86 were AHSs, and 54 were HNPs [37]. In 2014, Shaul et al. [38] reported the discovery of 327 persistent and bioaccumulative compounds in Southern California Bight common bottlenose dolphin (*Tursiops truncatus*) blubber, of which 180 were AHSs and 41 were NHPs. Two years later, Mackintosh et al. [39] identified 45 bioaccumulative DDT-related compounds in blubber from the same species. In 2019, Cossaboon et al. [36] reported a total of 194 halogenated contaminants in the blubber of five marine mammals (long-beaked common dolphin, short-beaked common dolphin, Risso’s dolphin, California sea lion, and Pacific harbor seal) from the Southern California Bight, including 30 HNPs. Most of the compounds (ca. 80%) detected in all these studies are typically not monitored.

Two studies involved marine mammals from Australia and South America. Hauler et al. [41] reported the identification of > 400 polyhalogenated compounds in dolphin (*Sousa chinensis*) blubber samples from Australia, many of which originated from unknown compounds. Additionally, Alonso et al. [40] detected 158 halogenated contaminants (including DDTs, MeO-BDEs, bromophenols, and mirex/dechloranes) in the blubber of bottlenose dolphins (*Tursiops truncatus*) from Rio de Janeiro, Brazil.

Fewer comprehensive screening studies have addressed the lower trophic levels of ecosystems. In 2015, Dwiyitno et al. [71] identified over 60 individual organic compounds including DDTs, high molecular weight polycyclic aromatic hydrocarbons (HMW-PAHs), and several emerging contaminants in six species of demersal and 2 pelagic fishes, banana shrimp (*Penaeus merguiensis*), and green mussel (*Perna viridis*) species from Jakarta Bay. According to the authors, some of these emerging contaminants had never previously been reported in Asian waters, namely diisopropylnaphthalenes (DIPNs), dichlorobenzene, DDMU, and phenylmethoxynaphthalene. Earlier this year, Goto et al. [42] conducted a study in the Asia-Pacific region and found ca. 60 halogenated contaminants (7 OCPs, 36 PCBs, 2 MeO-PBDEs, and 12 mixed halogenated compounds) in mussel (*Mytilus galloprovincialis*) samples from Hiroshima Bay, Japan.

Most of the abovementioned contaminant classes were detected in the samples studied in this work (cf. “Results and discussion” section; Tables 2, 3, and 4; Figs. 5 and 6; and ESM Figs. S1 and S2). Unfortunately, the studies cited above mainly focused on apex predator species and thus did not generate BMF values, preventing direct comparisons.

Losada et al. [72] investigated the biomagnification of anthropogenic and naturally produced organobrominated compounds in a marine food web in Sydney Harbour, Australia, and reported TMFs of 3.9, 3.3, 2.4, and 0.9 for summed PBDEs, 2′-MeO-BDE-68, 6-MeO-BDE-47, and TBA, respectively. MHC-1 was also biomagnified, but no reliable TMFs could be calculated due to the low detection frequency of this compound in fish samples. The MeO-BDEs exhibited similar biomagnification to the PBDEs and higher biomagnification than TBA, which was also the case in the current study (Table 4).

Additionally, geographic and tissue distribution studies have demonstrated the global distribution and biomagnification of halogenated DBPs [73] and MBPs [74], respectively, but no BMFs were reported.

## Conclusions

This work presents a new experimental workflow for non-selective extraction, purification (lipid removal), and non-target GC-HRMS screening to identify a wide range of lipophilic organic contaminants in biological samples. During GC-HRMS analysis, ECNI was used in addition to EI because of its higher sensitivity and specificity for halogenated compounds. The experimental workflow was complemented with separate data processing workflows for EI and ECNI data. Using these two workflows, BMF values were determined for legacy, emerging, and new contaminants in the species of the food webs in the Baltic Sea.

The results obtained show that a wide variety of contaminants accumulate and biomagnify in the tissues of Baltic Sea species. BMFs were calculated for all contaminants that occur throughout the food web or in specific predator-prey pairs. Applying a BMF cutoff value made it possible to considerably reduce the number of compounds needing to be identified.

As expected, the identified biomagnifying compounds included several legacy POPs that exhibited the expected biomagnification properties in the Baltic Sea food web, as shown in Fig. 7 (for *p*,*p*′-DDE and CB-153). The BMF values determined for these compounds agreed well with those reported in the literature, demonstrating that the sample preparation (extraction, clean-up, and fractionation), instrumental analysis, and data processing workflows all worked well.

However, in the future, it may be desirable to replace gravity flow Florisil^®^ columns with an alternative fractionation system such as semi-preparative high-performance liquid chromatography (HPLC) to separate PCBs and other contaminants present at relatively high concentrations from less abundant emerging contaminants. To further reduce the risk of co-elution, comprehensive two-dimensional GC (GC×GC) could be used instead of GC. This technique has previously been used together with LRMS in similar studies [24, 36–40, 43]. The combination of GC×GC and HRMS should provide outstanding separation power and facilitate identification of biomagnifying emerging contaminants.

As regards data processing, the top-down approach used in the ECNI workflow presented here could also be applied to EI data in the future. This may avoid loss of features with low signal quality in datasets for lower trophic level species during data processing and filtering. However, top-down approaches may be time consuming if applied to multiple species. It therefore seems better to focus on tissues from top consumer species that are rich in contaminants, e.g., harbor porpoise blubber, and use the resulting data for custom library development. It is expected that more compounds of interest will be found by combining the multi-species and top-down data processing workflows than by using a single NTS data processing workflow.

Although GC-TOF/MS is well suited for many lipophilic compounds, some potentially biomagnifying acidic, semi-polar, and polar AHSs, including various pharmaceuticals, may be lost during analysis. To widen the scope of future studies, derivatization may be considered. However, most derivatives are unstable [30, 71] and interfering compounds may be introduced or formed upon derivatization, which may hamper data processing and identification. It may therefore be preferable to determine AHSs of these types using HPLC-HRMS [30]. Such analyses are also likely to capture metabolites of lipophilic AHSs and HNPs.

## Electronic supplementary material

ESM 1 (DOCX 542 kb).

## Data Availability

The datasets generated during and/or analyzed during the current study are available from the corresponding author on reasonable request.
