# Supplementary material for: Non-targeted screening workflows for gas chromatography–high-resolution mass spectrometry analysis and identification of biomagnifying contaminants in biota samples
Source: Anal Bioanal Chem. 2020 Nov 6;413(2):479–501. doi: 10.1007/s00216-020-03018-4 (PMC7806533; doi:10.1007/s00216-020-03018-4)
Supplement: Supplementary file 1 — (DOCX 542 kb). [file 216_2020_3018_MOESM1_ESM.docx]

**Analytical and Bioanalytical Chemistry**

**Electronic Supplementary Material**

**Non-targeted screening workflows for gas chromatography – high-resolution mass spectrometry analysis and identification of biomagnifying contaminants in biota samples**

Andriy Rebryk and Peter Haglund

Department of Chemistry, Umeå University, Chemical Biological Centre (KBC), Linnaeus väg 6, 901 87 Umeå, Sweden

[andriy.rebryk@umu.se](mailto:andriy.rebryk@umu.se)

**Fig. S1** Total ion chromatograms (TIC) from GC-EI-HRMS analysis of the three Florisil^®^ fractions of a herring sample for the retention time range 12-42 min. (**a**) Florisil^®^ fraction 1, (**b**) Florisil^®^ fraction 2, (**c**) Florisil^®^ fraction 3. The most abundant biomagnifying compounds, the internal standard (Phenanthrene-D10), and the most abundant naturally occurring compounds are indicated. System peaks and compounds present in blanks are also included, and are labelled with an asterisk *


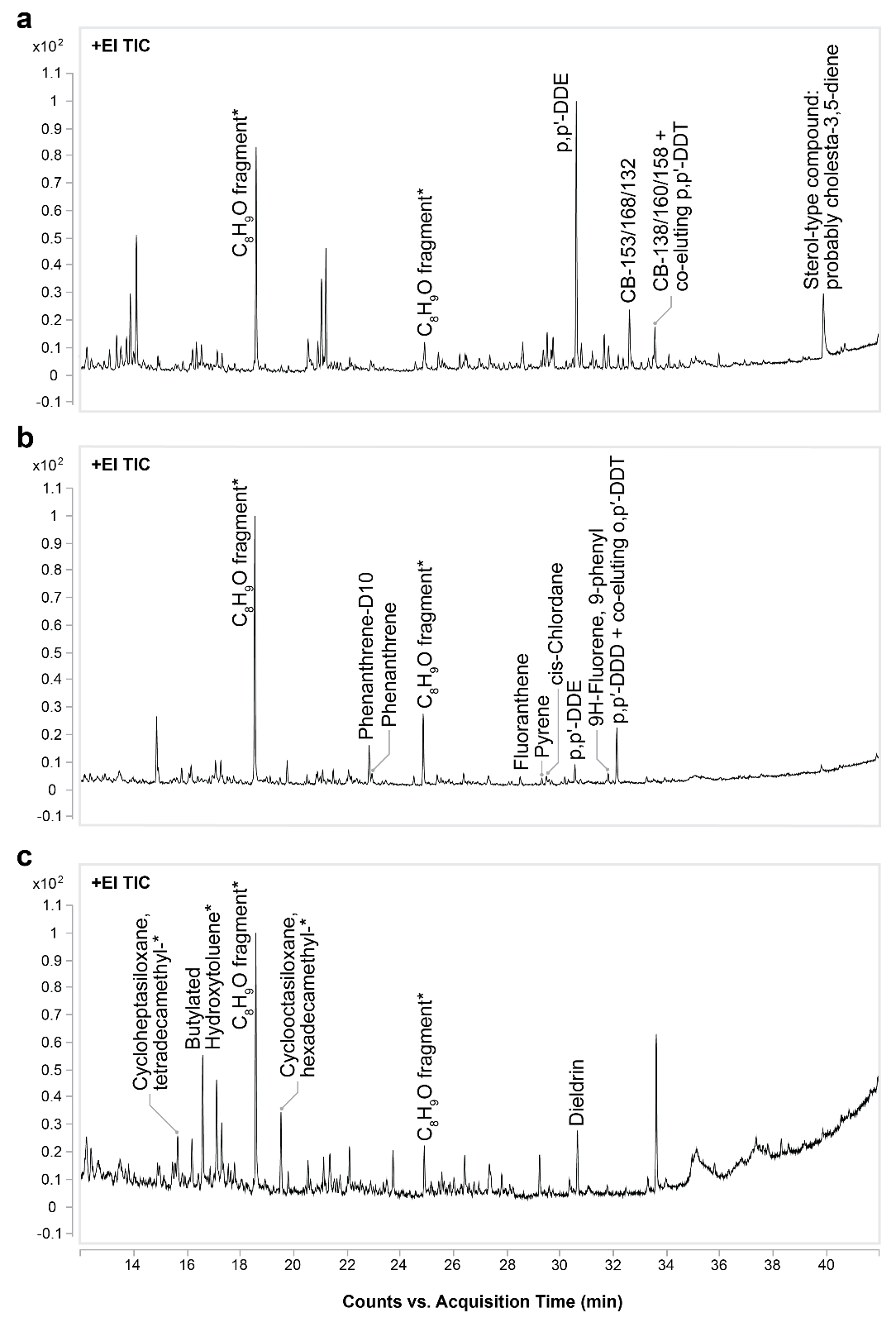


**Fig. S2** Typical EI spectrum from baseline regions of the TIC shown in Fig. 6A


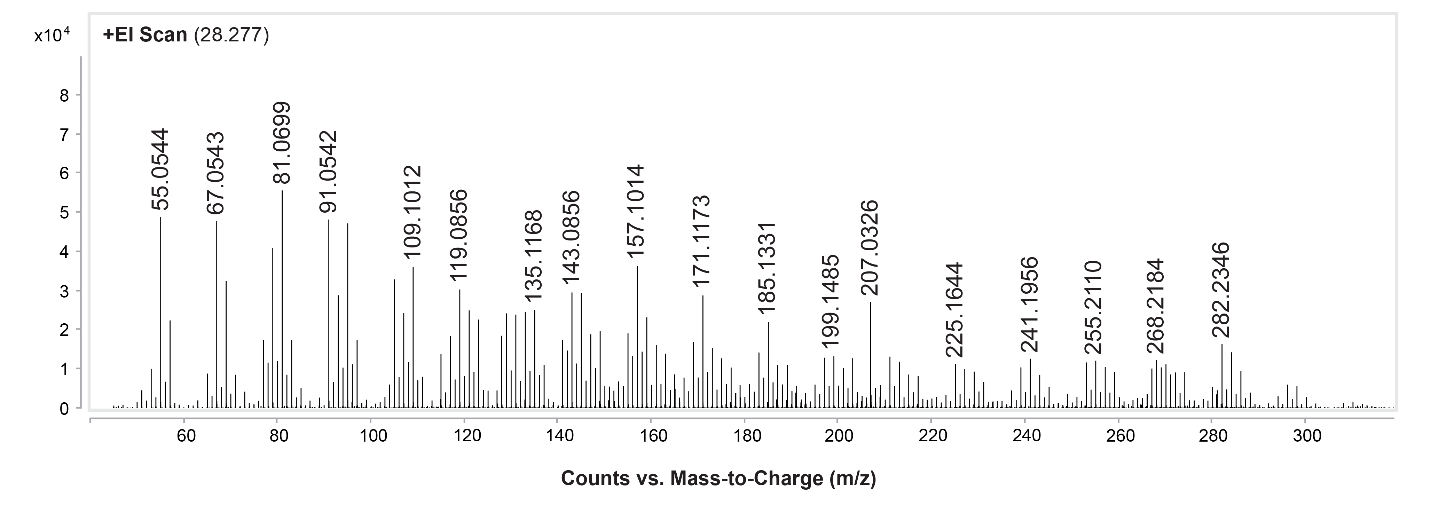


**Workflow S1** Agilent Unknowns Analysis & TOF Quantitative Analysis Workflow

1. Agilent Unknowns Analysis (UA) custom method development and reference (template) sample analysis to create custom libraries based on the search results
2. Open UA and create new analysis file to location of reference samples pressing New Analysis.
3. Add one of each reference sample instrument triplicate files to the new analysis using Add Samples. Herring muscle (HM) & eelpout muscle (EM) samples are chosen as reference samples (the samples for libraries templates)
4. Convert samples to SureMass: Tools – Convert Accurate Mass Samples – Convert to SureMass format – Select All; unselect Convert to TDA format. Press Convert. Close when done. Save analysis
5. Create custom method: press Edit Method and set the following parameters:
   - In Peak Detection choose SureMass
   - In Peak Detection – Peak filter set SNR threshold to 0
   - In Deconvolution – Resolution set RT window size factor to 25, 50, 100, 200
   - In Deconvolution – Ion peaks set Min # of ion peaks to 3 and Max # of ion peaks to 10
   - In Library Search – Libraries choose the library file, e.g., NIST17.L for NIST’17 spectral library used in this case
   - In Library Search – Match Factor – Use RT match: Gaussian – 6 s
   - In Library Search – RT mismatch penalty – Additive – Max RT penalty: 20
   - In Library Search – RT calibration file: e.g., RTI_DATA.csv (includes 4 columns: compound names, CAS numbers, retention indices (RI), and retention times (RT) of several well-known compounds, see Table S1)
   - In Compound Identification set Min match factor to 60 and Min MZ to 45
   - In Target Match – Target requirements select Target response and Qualifier ion(s)
   - In Target Match – Hit ion match criteria select Target ion and Qualifier ion(s)
   - In Target Match – Hit RT match criteria select Within target RT window
   - In Advanced settings set Min peak shape quality to 60. Apply and return to Standard
   - Press Apply to All Samples, Close and Save Method
6. Press Analyze All to run reference samples search. Save when done
7. Create custom libraries
8. Create a UA components table layout (right mouse click – Add/Remove Columns), which includes at least the following columns: Component RT, Compound Name, Match Factor, Component Area, Base Peak Area, Base Peak SNR (signal-to-noise ratio), Library RI, Hit RI, Delta RI (ΔRI), Library File. Save layout: press View – Load/Save Layout – Save Layout
9. Use HM & EM UA search results as a template for the custom library. In UA do the following:
   - In Component Filter, pick Hit for components that will be exported to “hits” library, which will contain components with assigned names from NIST’17 library
   - Right mouse click on Components table – Export – Export from: All components/hits
   - Export to: Library
   - Prefix: Reference + Add RT – OK
   - Library Editor (LE) will be opened – give the library a name with suffix “_0”, which means that it is a raw unedited library; save the library in Library Editor
   - Then in UA Component Filter pick Non-Hit for components that will be exported to “non-hits” libraries (will contains all other components) and repeat previous 4 steps
10. Create copies of the custom libraries “_0” (this step is done to save every previous version of respective library from editing for comparison if needed), rename them using, e.g., suffix “_1”, open them in Library Editor. For “non-hits” libraries copy Compound Names (e.g., Reference 32.6523) to CAS# column. Then create unique name for each of replicate compounds by adding a suffix, e.g., Tetratriacontane, Tetratriacontane_2, etc. Save. Or, instead of manual renaming that can take several hours depending on the number of components, use Python Script S1 (“hits”) or Script S2 (“non-hits”). The two scripts are slightly different, since “non-hits” library contains less rows, e.g., it doesn’t contain row with boiling point information, as it is unknown compound, so no boiling point is available
11. Run UA search of triplicates of reference samples again using both “_1” custom libraries in parallel; export hits of one of each reference sample (one of the triplicates used in the previous steps) searched using respective library (e.g., EM library for EM sample) as the new custom libraries with a suffix “_VL”, which means VLOOKUP (see the coming step)
12. Analyze triplicates of both HM & EM reference samples in parallel in MS Excel sheet using VLOOKUP function to sort out all the “noise” compounds, compounds detected in 1 sample out of 3 only
13. Copy Component table results from the last UA search to MS Excel. Save file
14. Use one sample of each triplicate as a template for VLOOKUP comparison (sample from sub-step g.). Use Component areas for VLOOKUP comparison
15. VLOOKUP function example: =VLOOKUP(AD5, D$5:H$250, 5, FALSE), where AD5 – cell with a name of compound in the reference sample used for comparison, e.g., sample from sub-step g., D$5 – cell with a name of compound in another sample, H$250 – component peak area in another sample, 5 – distance from name cell to area cell in another sample. Create template Excel file for other samples.
16. In LE, remove all compounds found only in one sample from the “_VL” libraries. Or, instead of manual renaming that can take several hours depending on the number of components, use Python Script S3 (“hits”) or Script S4 (“non-hits”). The two scripts are slightly different, since “non-hits” library contains less rows, e.g., it doesn’t contain row with boiling point information, as it is unknown compound, so no boiling point is available
17. Analyze triplicates of procedure (Florisil^®^) and solvent blanks (isooctane) & perform blank filtering
18. Run the UA search with the same parameters and layout for blanks; use both HM & EM “_VL” libraries in parallel
19. Copy Component table results from UA to MS Excel. Save file
20. Copy Component table results from UA for EM & HM triplicates to the same file
21. Run the UA search for reference samples, but using respective “_VL” custom libraries. Copy Component table results from UA to the same file and use it as a template for VLOOKUP comparison for blank filtering
22. Calculate median Component area for each component in each sample; then calculate ratio Sample : Isooctane & Sample : Florisil^®^ blank in MS Excel
23. Filter out the compounds for which Component areas of the samples are less than 5x Isooctane & Florisil^®^ blanks Component areas
24. Edit & finalize custom libraries
25. Create copies of “_VL” libraries and rename them using suffix “_Final”
26. In LE, remove all compounds for which the ratios are less than 5 during Blank filtering step from the “_Final” libraries. Or, instead of manual renaming that can take several hours depending on the number of components, use Python Script S5 (“hits”) or Script S6 (“non-hits”). The two scripts are slightly different, since “non-hits” library contains less rows, e.g., it doesn’t contain row with boiling point information, as it is unknown compound, so no boiling point is available
27. Agilent TOF Quantitative Analysis (TOFQA) method development
28. Open TOFQA and create a batch to location of reference samples. In this workflow reference samples are treated as calibration samples. Add reference samples to the batch. Save the batch
29. Start creating method from reference custom library file: Method – New – New Method from Library – Select reference custom library file:
    - In Targets section select All compounds
    - In Spectra section select Create targets per spectrum in the library
    - In Target ion section select Weighted
    - In Retention time calibration section select Use RT calibration – Browse – Upload respective .csv file with RI data (e.g., RTI_DATA.csv, Table S1)
    - In Qualifiers section set Number of Qualifiers to add value to 2. Click OK when done
30. Start refining the method from Method Setup Tasks section:
    - In Compound Setup section set Scan to Scan
    - Specify target peak selection criteria: Close RT or Close RT with Qualifiers are recommended
    - In ISTD Setup set one or few compounds as internal standard (ISTD) and then use it/those for any other compound
    - In Concentration Setup set concentration to one (1) for any compound and then copy it across all other compounds
    - In Calibration Curve Setup set CF (curve fit) to Average of Response Factors and CF Origin to Include
    - In Qualifier Setup change Uncertainty to 30 and Relative (complies with SANTE guidelines)
    - In Globals Setup set Reference Window to 0.5 and Reference Window Type to Minutes; Non Reference Window to 0.2 and Non Reference Window Type to Minutes. Correlation Window defines how much qualifier peak RT can differ from quantifier peak RT in minutes and still be regarded as qualifier for the target: change value to 0.1. Enable SureMass data processing
31. In Outlier Setup task user can specify different outliers that help reviewing data in TOFQA:
    - Set Sample Amount outlier values to 0.5 and 2 respectively to highlight fold change information when component is found in sample to be less than half or more than twice compared to reference samples
    - In Mass Accuracy set Mass Accuracy Limit to 5 ppm (complies with SANTE guidelines)
32. Advanced Tasks:
    - In Mass Extraction Setup set extraction windows (Extract Right m/z & Extract Left m/z) to 10 ppm for all quantifiers and qualifiers (use Right mouse click – Fill Down to copy values for all compounds)
    - Validate the method. Correct method errors, if any (error window will appear)
    - Save as
    - Exit the method editor and apply method to a batch. Select None (additional processing steps can be done later), click Yes and Save the batch before processing
33. Press Analyze Batch to analyze Reference samples
34. Review all items that have outliers highlighted (errors might occur due to bad integration (change integration parameters) and/or noisy signal (change quantifier/qualifier to different m/z))
35. Select sample and run Update Qualifier Ratios to update ratios using selected sample
36. Set Reference Samples Type to Cal and Level (1) as specified in the method
37. Run Analyze Batch again. Calibration curve will get updated
38. TOFQA sample analysis
39. Create a batch to location of the samples. Add all samples (in triplicates), incl. reference samples, to the batch. Save the batch
40. Analyze all samples in TOFQA in a similar way to p. 6, sub-steps y–cc. Save Batch with all samples
41. Analyze all samples in UA
42. Import EM & HM Quant results to parallel UA searches: File – Import Quantitative Analysis
43. In Method, change library to the respective “_Final” HM & EM libraries
44. Run Analyze All
45. Filter the results
46. After UA search, copy the result tables (Target Matches) of each sample triplicates to respective MS Excel files and perform component filtering using ±10 RI units (ΔRI = Library RI - Hit RI) – Step 1 (tab 1 in respective sample MS Excel file)
47. Copy the components for which ΔRI = ±10 to a new table, and do VLOOKUP for triplicate samples – Step 2 (tab 2 in respective sample MS Excel file)
48. Use one sample of each triplicate as a template for VLOOKUP comparison
49. Find geometrical mean (GEOMEAN) for the components detected in 2 or 3 samples.
50. Copy components that are left to Final table (important table columns: RT, Compound name, Component area GEOMEAN)
51. Copy Final tables of each sample to one MS Excel file. Save file
52. Recalculate Component areas to 1 g of sample (normalized Component areas to 1 g of fat)
53. Perform VLOOKUP step to find out which compounds are present in all samples. Use HM as a template for VLOOKUP comparison
54. Copy components found in all samples to a new table
55. Calculate BMFs in MS Excel
56. Copy MS Excel page with all samples and create tables for calculation of BMFs:

$$BMF = \frac{C(predator)}{C(prey)} \sim\frac{AR(predator)}{AR(prey)}$$

 ,

where C denotes the concentration of a given component in a predator and a prey sample, and *AR* is the ratio of the component’s area to that of the (closest eluting) volumetric standard, which is proportional to its concentration. Since no quantification standards have been used, the BMF values were calculated using component area ratios. This still yields valid BMFs as the analyte responses are instrument dependent, not sample dependent. To enhance robustness, the BMFs were calculated using the geometric means of the AR of the triplicate GC-MS runs. For compounds that were found in more than one Florisil^®^ fraction, the sum of the ARs were used in the calculations.

1. Use blue mussel flesh and herring muscle (Component area in 1 g of fat) as templates for BMFs calculation for lower trophic level consumers and top consumers, respectively
2. Calculate BMFs for specific predator:prey pairs, e.g., porpoise blubber:herring muscle
3. Repeat the whole procedure for other Florisil^®^ fraction
4. Finalize the data from all fractions

**Table S1** Retention index/retention time data (RTI_DATA.csv)

| Compound name | CAS # | Retention index | Retention time |
| --- | --- | --- | --- |
| D5 | 541-02-6 | 1034 | 6.87 |
| Naphthalene | 91-20-3 | 1162 | 8.523 |
| Dodecane | 112-40-3 | 1200 | 8.939 |
| D6 | 540-97-6 | 1351 | 11.535 |
| Biphenyl | 92-52-4 | 1366 | 13.704 |
| Tetradecane | 629-59-4 | 1400 | 14.255 |
| Pentadecane | 629-62-9 | 1500 | 16.666 |
| PeCB | 608-93-5 | 1501 | 16.908 |
| Dibenzofuran | 132-64-9 | 1504 | 17.05 |
| Hexadecane | 544-76-3 | 1600 | 19.395 |
| HCB | 118-74-1 | 1691 | 21.247 |
| Heptadecane | 629-78-7 | 1700 | 21.572 |
| Octadecane | 593-45-3 | 1800 | 23.041 |
| D10-Phenanthrene | 1517-22-2 | 1784 | 23.266 |
| Dibutyl phthalate | 84-74-2 | 1924 | 26.821 |
| DDMU | 1022-22-6 | 2087 | 29.708 |
| CB101 | 37680-73-2 | 2117 | 29.716 |
| trans-Nonachlor | 39765-80-5 | 2127 | 29.8 |
| DDE | 72-55-9 | 2168 | 31.16 |
| CB153 | 35065-27-1 | 2300 | 32.653 |
| CB138 | 35065-28-2 | 2345 | 33.546 |
| CB180 | 35065-29-3 | 2484 | 36.008 |
| CB170 | 35065-30-6 | 2532 | 36.976 |
| Mirex | 2385-85-5 | 2535 | 37.259 |
| CB209 | 2051-24-3 | 2818 | 41.356 |
| Cholestra35diene | 747-90-0 | 2880 | 41.907 |

**Script S1** Python script for duplicates renaming in the “hits” libraries

Project Jupyter Notebook (<https://jupyter.org/>) was used to run the script.

To run the script do the following:

1. Create respective main folder (e.g., 01_Duplicates_Renaming_Hits) and copy in library to be edited in .xml format
2. Specify path to your folder, e.g., C:/Scripts/01_Duplicates_Renaming_Hits
3. Run Project Jupyter Notebook. New window will be opened in web browser
4. Choose New – Python 2 and copy in script
5. Press Run. New sub-folder called Output will be created in main folder to which resulting edited library will be saved
6. Duplicates and their quantity are shown as part of quality control

import os

import glob

from lxml import etree as ET

import codecs

folder = 'C:/Scripts/01_Duplicates_Renaming_Hits' #example of the path to main folder

os.chdir(folder)

if not os.path.isdir('Output'):

os.mkdir('Output') #creates new output sub-folder in main folder

parser = ET.XMLParser(recover=True)

parsed_file = ET.parse((glob.glob('*.xml')[0]), parser=parser)

root = parsed_file.getroot()

print 'Root length: '

print str(len(root)) + ' element(s)'

print '*'*115

print 'Root[0] length: '

print str(len(root[0])) + ' element(s)'

print '*'*115

allNames = []

repeated = {}

repeated_accounted = {}

for i in range(len(root)):

if not '}Library' in root[i].tag:

if 'CompoundName' in root[i][4].tag:

txt = root[i][4].text

allNames.append(txt)

else:

pass

for name in allNames:

if allNames.count(name)>1:

repeated[name] = allNames.count(name)

repeated_accounted[name] = 0

print 'Duplicates & quantity:'

print repeated

print '*'*115

for i in range(len(root)):

if not '}Library' in root[i].tag:

if 'CompoundName' in root[i][4].tag:

txt = root[i][4].text

if txt in repeated.keys():

if repeated_accounted[txt]<>0:

root[i][4].text = txt + '_' + str(repeated_accounted[txt]+1)

repeated_accounted[txt] = repeated_accounted[txt] + 1

else:

pass

else:

pass

parsed_file.write('Output/_1.mslibrary.xml', encoding='utf-8', xml_declaration=True)

print 'Done!'

**Script S2** Python script for duplicates renaming in the “non-hits” libraries

Project Jupyter Notebook (<https://jupyter.org/>) was used to run the script.

To run the script do the following:

1. Create respective main folder (e.g., 01_Duplicates_Renaming_Non-hits) and copy in library to be edited in .xml format
2. Specify path to your folder, e.g., C:/Scripts/01_Duplicates_Renaming_Hits
3. Run Project Jupyter Notebook. New window will be opened in web browser
4. Choose New – Python 2 and copy in script
5. Press Run. New sub-folder called Output will be created in main folder to which resulting edited library will be saved
6. Duplicates and their quantity are shown as part of quality control

import os

import glob

from lxml import etree as ET

import codecs

folder = 'C:/Scripts/01_Duplicates_Renaming_Non-hits' #example of the path to main folder

os.chdir(folder)

if not os.path.isdir('Output'):

os.mkdir('Output') #creates new output sub-folder in main folder

parser = ET.XMLParser(recover=True)

parsed_file = ET.parse((glob.glob('*.xml')[0]), parser=parser)

root = parsed_file.getroot()

print 'Root length: '

print str(len(root)) + ' element(s)'

print '*'*115

print 'Root[0] length: '

print str(len(root[0])) + ' element(s)'

print '*'*115

allNames = []

repeated = {}

repeated_accounted = {}

for i in range(len(root)):

if 'CompoundName' in root[i][2].tag:

txt = root[i][2].text

allNames.append(txt)

else:

pass

for name in allNames:

if allNames.count(name)>1:

repeated[name] = allNames.count(name)

repeated_accounted[name] = 0

print 'Duplicates & quantity:'

print repeated

print '*'*115

for i in range(len(root)):

if 'CompoundName' in root[i][2].tag:

txt = root[i][2].text

if txt in repeated.keys():

if repeated_accounted[txt]<>0:

root[i][2].text = txt + '_' + str(repeated_accounted[txt]+1)

repeated_accounted[txt] = repeated_accounted[txt] + 1

else:

pass

else:

pass

parsed_file.write('Output/_VL.mslibrary.xml', encoding='utf-8', xml_declaration=True)

print 'Done!'

**Script S3** Python script for deleting the filtered out features (1 out 3) from the “hits” libraries

Project Jupyter Notebook (<https://jupyter.org/>) was used to run the script.

To run the script do the following:

1. Create respective main folder (e.g., 02_VLOOKUP_Hits) and copy in library to be edited in .xml format and MS Excel file in .xlsx format in which VLOOKUP comparison was done. Script targets column with Compound Name in MS Excel file (e.g., column BH (or 59), see the following script): adjust the column number according to your MS Excel file, count starts from 0 not 1
2. Specify path to your folder, e.g., C:/Scripts/02_VLOOKUP_Hits
3. Run Project Jupyter Notebook. New window will be opened in web browser
4. Choose New – Python 2 and copy in script
5. Press Run. New sub-folder called Output will be created in main folder to which resulting edited library will be saved
6. Compound names and spectra deleted are shown as part of quality control

import os

import glob

from xlrd import open_workbook

from lxml import etree as ET

folder = 'C:/Scripts/02_VLOOKUP_Hits' #example of the path to main folder

os.chdir(folder)

if not os.path.isdir('Output'):

os.mkdir('Output') #creates new output sub-folder in main folder

xls_file = glob.glob('*.xlsx')[0]

book = open_workbook(xls_file,on_demand=True)

worksheet = book.sheet_by_index(0)

arrayofvalues = worksheet.col_values(59) #59 (BH in letter code) is a Compound name column number in result table in MS Excel file where VLOOKUP comparison was done; adjust according to yours; count start from 0 not 1

for value in arrayofvalues:

pass

parser = ET.XMLParser(recover=True)

parsed_file = ET.parse((glob.glob('*.xml')[0]), parser=parser)

root = parsed_file.getroot()

print 'Root length: '

print str(len(root)) + ' element(s)'

print '*'*115

print 'Root[0] length: '

print str(len(root[0])) + ' element(s)'

print '*'*115

allNames = []

allIDNames = []

for i in range(len(root)):

if not '}Library' in root[i].tag:

if 'CompoundName' in root[i][4].tag:

txt = root[i][4].text

txt_ID = root[i][1].text

allNames.append(txt)

allIDNames.append(txt_ID)

else:

pass

namesToDelete = []

IDsToDelete = []

for i in range(len(allNames)):

if not (allNames[i] in arrayofvalues):

namesToDelete.append(allNames[i])

IDsToDelete.append(allIDNames[i])

for i in range(len(allNames)):

if not (allNames[i] in arrayofvalues):

namesToDelete.append(allNames[i])

IDsToDelete.append(allIDNames[i])

namesDeleted = []

IDsdeleted = []

for i in range(len(root)):

if not '}Library' in root[i].tag:

try:

if 'Compound' in root[i].tag:

txt = root[i][4].text

txt_ID = root[i][1].text

if txt in namesToDelete:

namesDeleted.append(txt)

IDsdeleted.append(txt_ID)

else:

pass

except:

pass

print 'Compound Names deleted: '

print ' '

while len(namesDeleted)>0:

todo=1

for i in range(len(root)):

try:

if 'CompoundName' in root[i][4].tag:

txt = root[i][4].text

if txt in namesDeleted:

print txt

root.remove(root[i])

namesDeleted.remove(txt)

else:

pass

except:

pass

print '*'*115

IDspectraDeleted = []

for i in range(len(root)):

try:

if 'Spectrum' in root[i].tag:

txt_ID = root[i][1].text

if txt_ID in IDsToDelete:

IDspectraDeleted.append(txt_ID)

else:

pass

except:

pass

print 'Spectra deleted: '

print ' '

while len(IDspectraDeleted)>0:

todo=1

for i in range(len(root)):

try:

if 'Spectrum' in root[i].tag:

if 'CompoundID' in root[i][1].tag:

txt_ID = root[i][1].text

if txt_ID in IDspectraDeleted:

print txt_ID

root.remove(root[i])

IDspectraDeleted.remove(txt_ID)

else:

pass

else:

pass

except:

pass

print '*'*115

parsed_file.write('Output/_VL.mslibrary.xml', encoding='utf-8', xml_declaration=True)

print 'Done!'

**Script S4** Python script for deleting the filtered out features (1 out 3) from the “non-hits” libraries

Project Jupyter Notebook (<https://jupyter.org/>) was used to run the script.

To run the script do the following:

1. Create respective main folder (e.g., 02_VLOOKUP_Non-hits) and copy in library to be edited in .xml format and MS Excel file in .xlsx format in which VLOOKUP comparison was done. Script targets column with Compound Name in MS Excel file (e.g., column BH (or 59), see the following script): adjust the column number according to your MS Excel file, count starts from 0 not 1
2. Specify path to your folder, e.g., C:/Scripts/02_VLOOKUP_Non-hits
3. Run Project Jupyter Notebook. New window will be opened in web browser
4. Choose New – Python 2 and copy in script
5. Press Run. New sub-folder called Output will be created in main folder to which resulting edited library will be saved
6. Compound names and spectra deleted are shown as part of quality control

import os

import glob

from xlrd import open_workbook

from lxml import etree as ET

folder = 'C:/Scripts/02_VLOOKUP_Non-hits' #example of the path to main folder

os.chdir(folder)

if not os.path.isdir('Output'):

os.mkdir('Output') #creates new output sub-folder in main folder

xls_file = glob.glob('*.xlsx')[0]

book = open_workbook(xls_file,on_demand=True)

worksheet = book.sheet_by_index(0)

arrayofvalues = worksheet.col_values(59) #59 (BH in letter code) is a Compound name column number in result table in MS Excel file where VLOOKUP comparison was done; adjust according to yours; count start from 0 not 1

for value in arrayofvalues:

pass

parser = ET.XMLParser(recover=True)

parsed_file = ET.parse((glob.glob('*.xml')[0]), parser=parser)

root = parsed_file.getroot()

print 'Root length: '

print str(len(root)) + ' element(s)'

print '*'*115

print 'Root[0] length: '

print str(len(root[0])) + ' element(s)'

print '*'*115

allNames = []

allIDNames = []

for i in range(len(root)):

if 'CompoundName' in root[i][3].tag:

txt = root[i][3].text

txt_ID = root[i][1].text

allNames.append(txt)

allIDNames.append(txt_ID)

namesToDelete = []

IDsToDelete = []

for i in range(len(allNames)):

if not (allNames[i] in arrayofvalues):

namesToDelete.append(allNames[i])

IDsToDelete.append(allIDNames[i])

for i in range(len(allNames)):

if not (allNames[i] in arrayofvalues):

namesToDelete.append(allNames[i])

IDsToDelete.append(allIDNames[i])

namesDeleted = []

IDsdeleted = []

for i in range(len(root)):

try:

if 'Compound' in root[i].tag:

txt = root[i][3].text

txt_ID = root[i][1].text

if txt in namesToDelete:

namesDeleted.append(txt)

IDsdeleted.append(txt_ID)

else:

pass

except:

pass

print 'Compound Names deleted: '

print ' '

while len(namesDeleted)>0:

todo=1

for i in range(len(root)):

try:

if 'CompoundName' in root[i][3].tag:

txt = root[i][3].text

if txt in namesDeleted:

print txt

root.remove(root[i])

namesDeleted.remove(txt)

else:

pass

except:

pass

print '*'*115

IDspectraDeleted = []

for i in range(len(root)):

try:

if 'Spectrum' in root[i].tag:

txt_ID = root[i][1].text

if txt_ID in IDsToDelete:

IDspectraDeleted.append(txt_ID)

else:

pass

except:

pass

print 'Spectra deleted: '

print ' '

while len(IDspectraDeleted)>0:

todo=1

for i in range(len(root)):

try:

if 'Spectrum' in root[i].tag:

if 'CompoundID' in root[i][1].tag:

txt_ID = root[i][1].text

if txt_ID in IDspectraDeleted:

print txt_ID

root.remove(root[i])

IDspectraDeleted.remove(txt_ID)

else:

pass

else:

pass

except:

pass

print '*'*115

parsed_file.write('Output/_VL.mslibrary.xml', encoding='utf-8', xml_declaration=True)

print 'Done!'

**Script 5** Python script for deleting the features present in blanks at high level from the “hits” libraries

Project Jupyter Notebook (<https://jupyter.org/>) was used to run the script.

To run the script do the following:

1. Create respective main folder (e.g., 03_Blank_Filtering_Hits) and copy in library to be edited in .xml format and MS Excel file in .xlsx format in which blank filtering was done. Script targets columns with RT, Compound Name, Sample:Florisil^®^ and Sample:Isooctane ratios in MS Excel file (e.g., columns GC, GE, GF, GG (or 184, 186, 187, 188), see the following script): adjust the column numbers according to your MS Excel file, count starts from 0 not 1. The table values has to begin in row 1 of MS Excel file
2. Specify path to your folder, e.g., C:/Scripts/03_Blank_Filtering_Hits
3. Run Project Jupyter Notebook. New window will be opened in web browser
4. Choose New – Python 2 and copy in script
5. Press Run. New sub-folder called Output will be created in main folder to which resulting edited library will be saved
6. Compound IDs, RTs, Compound Names, and Spectra deleted are shown as part of quality control

import os

import glob

import math

from xlrd import open_workbook

from lxml import etree as ET

folder = 'C:/Scripts/03_Blank_Filtering_Hits' #example of the path to main folder

os.chdir(folder)

if not os.path.isdir('Output'):

os.mkdir('Output') #creates new output sub-folder in main folder

xls_file = glob.glob('*.xlsx')[0]

book = open_workbook(xls_file,on_demand=True)

worksheet = book.sheet_by_index(0)

excel_RT = worksheet.col_values(184) #RT in the final result table (count starts from 0)

excel_CompName = worksheet.col_values(186) #Compound name (count starts from 0)

excel_BlankRatio1 = worksheet.col_values(187) #Sample:Florisil ratio (count starts from 0)

excel_BlankRatio2 = worksheet.col_values(188) #Sample:Isooctane ratio (count starts from 0)

namesToDelete = []

RTsToDelete = []

print '*'*115

print 'RTs and Compound Names to be deleted (MS Excel): '

print ' '

for i in range(len(excel_RT)):

if excel_BlankRatio1[i]<5 or excel_BlankRatio2[i]<5:

print str(round(excel_RT[i], 3)) + ' ' + str(excel_CompName[i])

RTsToDelete.append(excel_RT[i])

namesToDelete.append(excel_CompName[i])

print '*'*115

print 'Number of compounds to be deleted (MS Excel): '

print ' '

print len(RTsToDelete)

print '*'*115

parser = ET.XMLParser(recover=True)

parsed_file = ET.parse((glob.glob('*.xml')[0]), parser=parser)

root = parsed_file.getroot()

allNames = []

allRTs = []

allIDs = []

IDsToDelete = []

print 'All Compound IDs, RTs and Compound Names in Library: '

print ' '

for i in range(len(root)):

if not '}Library' in root[i].tag:

if 'CompoundName' in root[i][4].tag:

CompName = root[i][4].text

CompRT = root[i][11].text

CompID = root[i][1].text

print str(CompID) + ' ' + str(round((float(CompRT)), 3)) + ' ' + str(CompName)

allNames.append(CompName)

allRTs.append(CompRT)

allIDs.append(CompID)

print '*'*115

namesDeleted = []

RTsdeleted = []

IDsdeleted = []

print 'Compound IDs, RTs and Compound Names deleted: '

print ' '

for i in range(len(root)):

try:

if 'Compound' in root[i].tag:

CompRT = root[i][11].text

CompName = root[i][4].text

CompID = root[i][1].text

if CompName in namesToDelete:

print CompID + ' ' + CompRT + ' ' + CompName

namesDeleted.append(CompName)

RTsdeleted.append(CompRT)

IDsdeleted.append(CompID)

else:

pass

except:

pass

print '*'*115

print 'Number of compounds in deleted list (Library): '

print ' '

print len(RTsdeleted)

print '*'*115

while len(namesDeleted)>0:

todo=1

for i in range(len(root)):

try:

if 'CompoundName' in root[i][4].tag:

CompName = root[i][4].text

if CompName in namesDeleted:

root.remove(root[i])

namesDeleted.remove(CompName)

else:

pass

except:

pass

IDspectraDeleted = []

for i in range(len(root)):

try:

if 'Spectrum' in root[i].tag:

CompID = root[i][1].text

if CompID in IDsdeleted:

IDspectraDeleted.append(CompID)

else:

pass

except:

pass

print 'Spectrum IDs deleted: '

print ' '

print sorted(IDspectraDeleted)

while len(IDspectraDeleted)>0:

todo=1

for i in range(len(root)):

try:

if 'Spectrum' in root[i].tag:

if 'CompoundID' in root[i][1].tag:

CompID = root[i][1].text

if CompID in IDspectraDeleted:

root.remove(root[i])

IDspectraDeleted.remove(CompID)

else:

pass

else:

pass

except:

pass

print '*'*115

parsed_file.write('Output/_Final.mslibrary.xml', encoding='utf-8', xml_declaration=True)

print 'Done!'

**Script 6** Python script for deleting the features present in blanks at high level from the “non-hits” libraries

Project Jupyter Notebook (<https://jupyter.org/>) was used to run the script.

To run the script do the following:

1. Create respective main folder (e.g., 03_Blank_Filtering_Non-hits) and copy in library to be edited in .xml format and MS Excel file in .xlsx format in which blank filtering was done. Script targets columns with RT, Compound Name, Sample:Florisil^®^ and Sample:Isooctane ratios in MS Excel file (e.g., columns GC, GE, GF, GG (or 184, 186, 187, 188), see the following script): adjust the column numbers according to your MS Excel file, count starts from 0 not 1. The table values has to begin in row 1 of MS Excel file
2. Specify path to your folder, e.g., C:/Scripts/03_Blank_Filtering_Non-hits
3. Run Project Jupyter Notebook. New window will be opened in web browser
4. Choose New – Python 2 and copy in script
5. Press Run. New sub-folder called Output will be created in main folder to which resulting edited library will be saved
6. Compound IDs, RTs, Compound Names, and Spectra deleted are shown as part of quality control

import os

import glob

import math

from xlrd import open_workbook

from lxml import etree as ET

folder = 'C:/Users/anre0098/Desktop/Software for NTDS wrkflw/Scripts - Test/03_Blank_Filtering_Non-hits' #example of the path to main folder

os.chdir(folder)

if not os.path.isdir('Output'):

os.mkdir('Output') #creates new output sub-folder in main folder

xls_file = glob.glob('*.xlsx')[0]

book = open_workbook(xls_file,on_demand=True)

worksheet = book.sheet_by_index(0)

excel_RT = worksheet.col_values(184) #RT in the final result table (count starts from 0)

excel_CompName = worksheet.col_values(186) #Compound name (count starts from 0)

excel_BlankRatio1 = worksheet.col_values(187) #Sample:Florisil ratio (count starts from 0)

excel_BlankRatio2 = worksheet.col_values(188) #Sample:Isooctane ratio (count starts from 0)

namesToDelete = []

RTsToDelete = []

print '*'*115

print 'RTs and Compound Names to be deleted (MS Excel): '

print ' '

for i in range(len(excel_RT)):

if excel_BlankRatio1[i]<5 or excel_BlankRatio2[i]<5:

print str(round(excel_RT[i], 3)) + ' ' + str(excel_CompName[i])

RTsToDelete.append(excel_RT[i])

namesToDelete.append(excel_CompName[i])

print '*'*115

print 'Number of compounds to be deleted (MS Excel): '

print ' '

print len(RTsToDelete)

print '*'*115

parser = ET.XMLParser(recover=True)

parsed_file = ET.parse((glob.glob('*.xml')[0]), parser=parser)

root = parsed_file.getroot()

allNames = []

allRTs = []

allIDs = []

IDsToDelete = []

print 'All Compound IDs, RTs and Compound Names in Library: '

print ' '

for i in range(len(root)):

if not '}Library' in root[i].tag:

if 'CompoundName' in root[i][3].tag:

CompName = root[i][3].text

CompRT = root[i][6].text

CompID = root[i][1].text

print str(CompID) + ' ' + str(round((float(CompRT)), 3)) + ' ' + str(CompName)

allNames.append(CompName)

allRTs.append(CompRT)

allIDs.append(CompID)

print '*'*115

namesDeleted = []

RTsdeleted = []

IDsdeleted = []

print 'Compound IDs, RTs and Compound Names deleted: '

print ' '

for i in range(len(root)):

try:

if 'Compound' in root[i].tag:

CompRT = root[i][6].text

CompName = root[i][3].text

CompID = root[i][1].text

if CompName in namesToDelete:

print CompID + ' ' + CompRT + ' ' + CompName

namesDeleted.append(CompName)

RTsdeleted.append(CompRT)

IDsdeleted.append(CompID)

else:

pass

except:

pass

print '*'*115

print 'Number of compounds in deleted list (Library): '

print ' '

print len(RTsdeleted)

print '*'*115

while len(namesDeleted)>0:

todo=1

for i in range(len(root)):

try:

if 'CompoundName' in root[i][3].tag:

CompName = root[i][3].text

if CompName in namesDeleted:

root.remove(root[i])

namesDeleted.remove(CompName)

else:

pass

except:

pass

IDspectraDeleted = []

for i in range(len(root)):

try:

if 'Spectrum' in root[i].tag:

CompID = root[i][1].text

if CompID in IDsdeleted:

IDspectraDeleted.append(CompID)

else:

pass

except:

pass

print 'Spectrum IDs deleted: '

print ' '

print sorted(IDspectraDeleted)

while len(IDspectraDeleted)>0:

todo=1

for i in range(len(root)):

try:

if 'Spectrum' in root[i].tag:

if 'CompoundID' in root[i][1].tag:

CompID = root[i][1].text

if CompID in IDspectraDeleted:

root.remove(root[i])

IDspectraDeleted.remove(CompID)

else:

pass

else:

pass

except:

pass

print '*'*115

parsed_file.write('Output/_Final.mslibrary.xml', encoding='utf-8', xml_declaration=True)

print 'Done!'
